# Deep empirical neural network for optical phase retrieval over a scattering medium

**DOI:** 10.1038/s41467-025-56522-5

**Published:** 2025-02-05

**Authors:** Huaisheng Tu, Haotian Liu, Tuqiang Pan, Wuping Xie, Zihao Ma, Fan Zhang, Pengbai Xu, Leiming Wu, Ou Xu, Yi Xu, Yuwen Qin

**Affiliations:** 1 Key Laboratory of Photonic Technology for Integrated Sensing and Communication, Ministry of Education, Guangdong University of Technology, Guangzhou, 510006 China; 2 Guangdong Provincial Key Laboratory of Information Photonics Technology, Guangdong University of Technology, Guangzhou, 510006 China; 3 School of Information Engineering, Guangdong University of Technology, Guangzhou, 510006 China; 4 Institute of Advanced Photonic Technology, Guangdong University of Technology, Guangzhou, 510006 China

**Keywords:** Optics and photonics, Optical techniques

## Abstract

Supervised learning, a popular tool in modern science and technology, thrives on huge amounts of labeled data. Physics-enhanced deep neural networks offer an effective solution to alleviate the data burden by incorporating an analytical model that interprets the underlying physical processes. However, it completely fails in tackling systems without analytical solution, where wave scattering systems with multiple input multiple output are typical examples. Herein, we propose a concept of deep empirical neural network (DENN) that is a hybridization of a deep neural network and an empirical model, which enables seeing through an opaque scattering medium in an untrained manner. The DENN does not rely on labeled data, all while delivering as high as 58% improvement in fidelity compared with the supervised learning using 30000 data pairs for achieving the same goal of optical phase retrieval. The DENN might shed new light on the applications of deep learning in physics, information science, biology, chemistry and beyond.

## Abstract (web summary)

Tu et al. demonstrate that the unique integration of a convolutional neural network with an empirical model measured for an optical scattering system can unscramble the scattering and see through the opaque medium in an untrained fashion, featured as a deep empirical neural network.

## Introduction

Deep learning (DL) has become a revolutionary paradigm for solving inverse problems in science and technology^1–3^, including physics^4–7^, biology^8,9^, chemistry^10,11^, information science^12^ and engineering^13,14^. As a typical type of DL, supervised learning excels at learning a complex relationship from large amounts of input and output data pairs for an unknown system in an end-to-end manner^15^, which has shown its capability in overcome critical challenges in computer vision^16,17^, natural language processing^18,19^, medical diagnosis^20–22^, and weather forecast^23,24^. In order to learn a mapping function of the unknown system, large-scale labeled data and extensive training are indispensable prerequisites^15^, which are time-consuming and energy-intensive. Unsupervised and self-supervised learning have been proposed to release the heavy effort of registering data pairs^25–31^, while sufficiently large amounts of data are still need.

Therefore, there is a growing pursuit of developing untrained deep neural networks (DNNs) for acquiring the mapping function of unknown systems, where physics-enhanced DNNs have been proposed to overcome the challenge of big data by integrating physical prior into the neural network^32–47^. Inspired by the concept of deep image prior (DIP)^48^, untrained neural networks have been widely used in metrology^4^, holography^36,42,43,46,47^, and imaging^29,39,40,44,45^. In particular, there are several variants of the DIP for optical phase retrieval in diffraction optics^33,34,39,49^, where physics consistency is forced by minimizing the loss function between the estimated results and the actual measurement. Typical DIP-type DNNs for solving this inverse problem rely on a forward mapping function^33,34^ to generate the estimated results, where an analytical model describing the physical evolution process is required^25,28,33^. The weights and biases of a physics-enhanced DNN can be updated using a loss function based on the analytical theory. It is quite efficient in dealing with the ill-posed problem of phase retrieval with solo intensity measurement^33,34,39,49–51^, where the phase retardation can be precisely described by a theory. However, generating the estimated results using an empirical model relied on the real response of system, where the model should be differentiable for the back propagation of the neural network, resembles a promising while open attempt to update the neural network in an untrained manner. Although beam propagation method^52^ can account for weak scattering scenarios with negligible backward scattering^53,54^, the analytical models can only be applied in the scenario where strongly optical scattering is absent^33,34^. Furthermore, there are many optical systems without analytical theory^55^, where wave scattering systems with non-negligible backward scattering are typical examples. It renders the physics-enhanced DNNs based on the analytical theory inapplicable in these scenarios. Therefore, leveraging the DIP concept for achieving optical phase retrieval over a scattering medium remains an alluring but challenging prospect.

Herein, we report a DENN for tackling scattering systems without analytical theory, where phase retrieval through an optical multiple input multiple output (MIMO) system is demonstrated by simulation and experiment, simultaneously. An experimentally calibrated empirical transmission matrix (TM) describing the forward mapping relationship of the MIMO system is integrated to the DNN for the back propagation of the neural network. This empirical model captures the relationship between multiple input and their corresponding output for the scattering system. However, unlike an analytical theory that can predict the full evolution of the system for any given input, this model resembles a partial experimental measurement of the system, providing insights for the update of DENN. It is demonstrated that the need of labeled data and training for the DENN can be eliminated by integrating the empirical model of the opaque system with a convnet.

As a proof of principle, seeing through a multimode fiber (MMF) and a ground glass are achieved using a single-shot speckle intensity at their output. Considering the supervised learning using 30000 data pairs, the training and inference time are about 26 hours and 0.48 second, respectively. While the DENN achieve the same goal of optical phase retrieval using the inference time of 270 seconds, all while delivering over 58% improvement in fidelity for a given natural scene image encoded information. Our quantitative simulation and experimental results verify the feasibility and superiority of the proposed DENN on solving the ill-posed inverse problem of phase retrieval using a single intensity input. Because the empirical TM can be calibrated for different scattering media, the combination of a neural network with the empirical TM is applicative for various media. Therefore, the DENN can be generalized for diverse inverse problems of information retrieval in black box systems without analytical theory.

## Results

### Principle

**Fig. 1:**
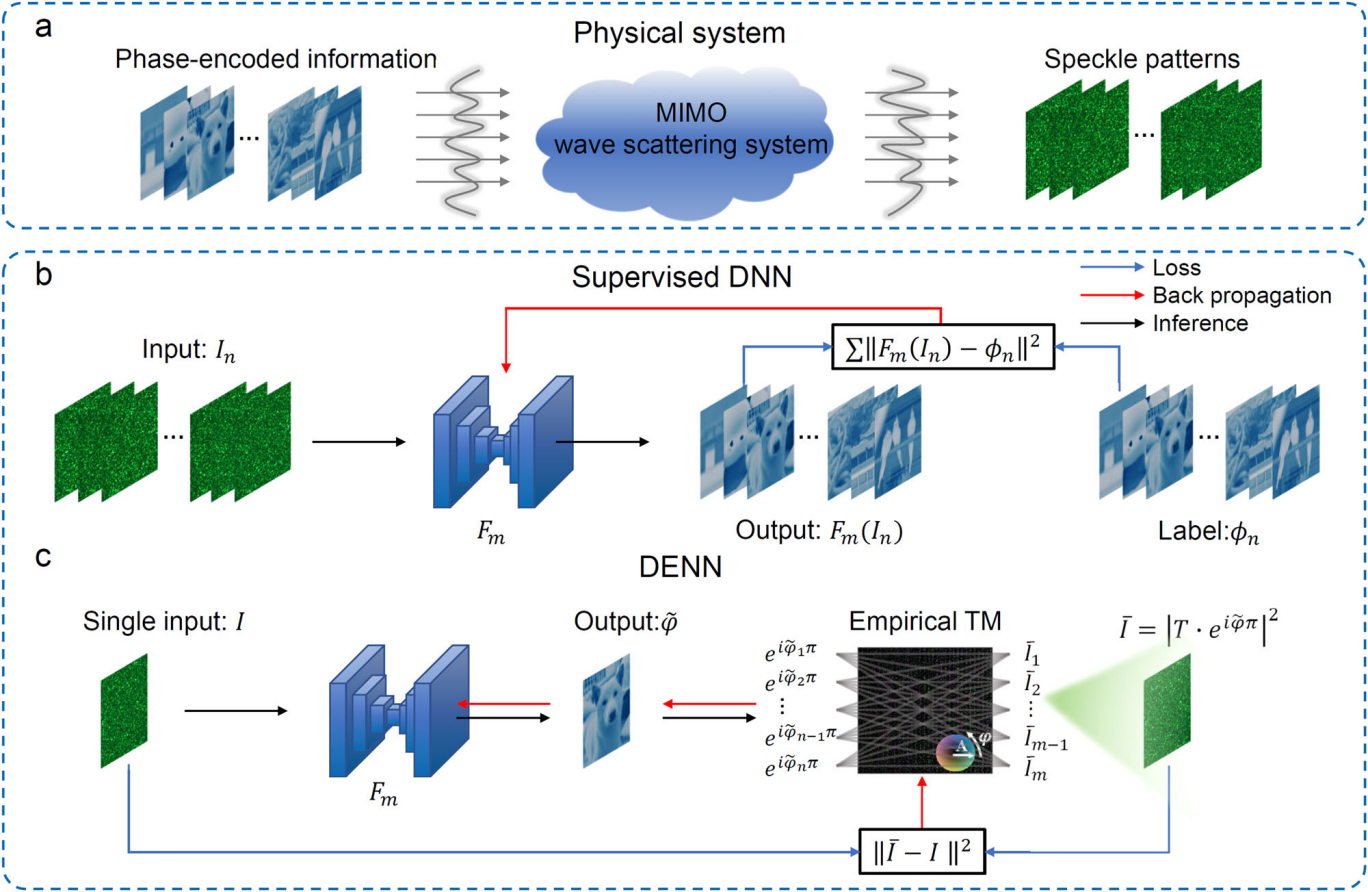
Schematics of the supervised deep neural network and the deep empirical neural network for a wave scattering system with multiple input and multiple output (MIMO). **a** A schematic of the MIMO wave scattering system. The chaotic speckle pattern is the output of the physical system, where phase-encoded input information is substantially scrambled. **b** A large number of speckle patterns *I*_*n*_ and their phase-encoded information are the input and output of a supervised deep neural network (DNN). The loss function between the output of the neural network ${F}_{m}\left({I}_{n}\right)$ and the ground truth of phase-encoded information *ϕ*_*n*_ is used to update the parameters of the neural network. **c** The input of the deep empirical neural network (DENN) only requires a single speckle pattern *I*. The DENN will produce an estimated phase $\widetilde{\varphi }$ at first, and then an estimated speckle pattern $\bar{I}$ will be produced by the empirical transmission matrix (TM). The loss function between the measured speckle pattern *I* and the empirical one $\bar{I}$ is used to update the parameters of the DENN. A typical empirical TM calibrated for an MMF is shown in the figure as an example, where its amplitude and phase are presented. Blue arrows in (**b**) and (**c**) indicate the calculation of loss function, red arrows show the gradient back propagation while black arrows indicate the inference of networks.

Considering a wave scattering system of MIMO property shown in Fig. 1a, multiple phase-encoded information in spatial division input channels is scrambled by the scattering system, resulting in multiple output information manifested itself as chaotic speckle pattern. A conventional supervised DNN for decoding phase information from a seemly chaotic speckle pattern relies on a large amount of labeled data in the training set^32,56–59^${D}_{S}=\left\{\left({\phi }_{n},{I}_{n}\right);n=1,\ldots,N\right\}$ by solving

1 $${F}_{{m}^{*}}=\arg {\min }_{m\in M}{\left\Vert {F}_{m}\left({I}_{n}\right)-{\phi }_{n}\right\Vert }^{2},\quad \forall ({\phi }_{n},{I}_{n})\in {D}_{S}$$

where *F*_*m*_ is the mapping function determined by the weights and biases of the DNN, as shown in Fig. 1b. Here, *m* ∈ *M* and *M* are all possible weights and biases of the network. After training, a feasible mapping function ${F}_{{m}^{*}}$ is obtained, which can map the speckle pattern *I* that is not in the training set *D*_*S*_ back to the corresponding information encoded in the phase dimension ${F}_{{m}^{*}}\left(I\right)$. However, tens of thousands of labeled data pairs are required, where the experimental registrations of such a mass of speckle patterns *I*_*n*_ and their corresponding ground truths of phase-encoded information *ϕ*_*n*_ are very time-consuming.

On the contrary, the proposed DENN for seeing through a wave scattering medium with MIMO property relies on the hybridization between an empirical TM and a convnet (see Supplementary Note 1 for the architecture of convolutional network). As shown in Fig. 1c, the DENN only needs a single speckle pattern *I* to retrieve the phase-encoded information $\widetilde{\varphi }$ scrambled by the scattering medium. Compared with the conventional DNNs, which strictly depend on the training dataset of phase-encoded information *ϕ*_*n*_ and the corresponding speckle patterns *I*_*n*_, the DENN does not require the ground truth of the single input speckle *I* for phase retrieval. Instead, it leverages the empirical TM to generate the virtual speckle pattern $\bar{I}$, which is then used for evaluating the value of loss function with the measured *I*. Essentially, the empirical TM for updating the DENN can be regarded as a fully connected (FC) layer with fixed weights right after the convnet, effectively describing the MIMO scattering process of the scattering medium. Therefore, it can be integrated into the topological structure of the DNN, as schematically indicated in Fig. 1c. Other weights and biases of the DENN can be updated through the AdamW optimizer^60^ (See Methods section for details). After iterative processing, it will force the retrieved phase dependent speckle pattern $\bar{I}$ to converge to the measured speckle pattern *I*, finalizing the update of neural network. Without loss of generality, the convnet can be other types of neural network.

To elaborate the process of the DENN, the empirical TM of an MMF is considered first as an example. In general, the input-output relationship of the MMF can be bridged by a TM^61^, as shown in the following formula:

2 $${E}_{{{\rm{out}}}}=T{E}_{in}$$

where *T* represents the empirical TM of the MMF^62^. And *E*_in_ and *E*_*out*_ are the corresponding input and output complex light fields, respectively. Therefore, the output speckle pattern recorded by a camera can be expressed as:

3 $$I={\left\vert {E}_{{{\rm{out}}}}\right\vert }^{2}={\left\vert T{E}_{in}\right\vert }^{2}$$

Because the phase-encoded information can be evaluated by $\widetilde{\varphi }={F}_{m}(I)$, the DENN can be updated for a single intensity input *I* utilizing

4 $${F}_{{m}^{*}}=\arg \min {\left\Vert \bar{I}-I\right\Vert }^{2}=\arg \min {\left\Vert | T{e}^{i\cdot \widetilde{\varphi }\cdot \pi }{| }^{2}-I\right\Vert }^{2}$$

where the empirical TM links the normalized phase-encoded information $\widetilde{\varphi }={F}_{m}(I)$ of multiple input with the speckle intensity $\bar{I}$ of multiple output. As shown in Eq. (4), the ground truth *φ* is not involved in the loss function, which means the DENN can be updated in an untrained manner. It is the unique combination of the empirical TM and the convnet that allows the DENN to capture the empirical knowledge of the wave scattering system. When the converged condition of Eq. (4) is met, the mapping function ${F}_{{m}^{*}}$ can be used to retrieve the phase-encoded information for the input speckle *I*:

5 $$\widetilde{\varphi }={F}_{{m}^{*}}(I)$$

It should be noted that seeing through an MMF is used as a typical example to demonstrate the superiority of the DENN for unscrambling the chaotic scattering of the strongly scattering medium. This concept can be generalized to other scattering media, whose TM can be calibrated (See Discussion section for details).

### Accuracy of the empirical model

**Fig. 2:**
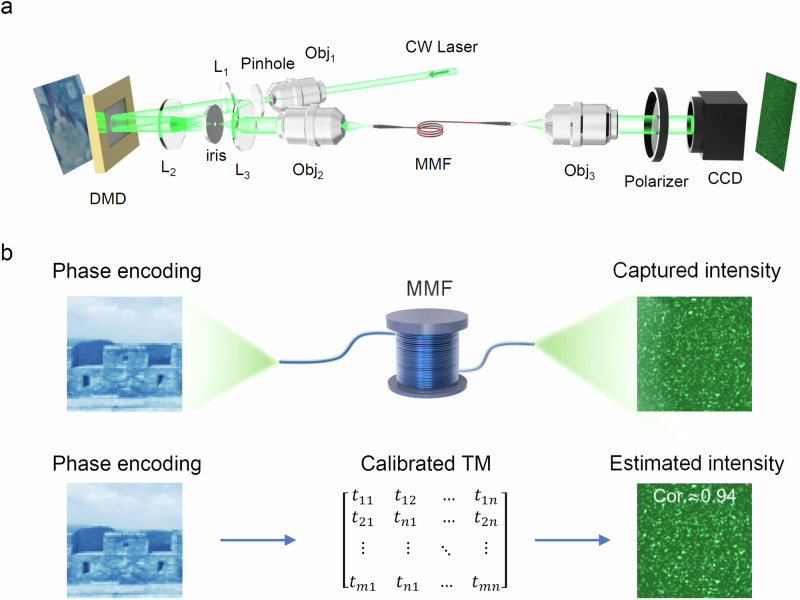
The accuracy of empirical model. **a** The optical setup for calibrating the empirical transmission matrix (TM) of the multimode fiber (MMF). Here, DMD is a digital micromirror device and CW laser indicates a continuous-wave laser. Obj denotes the microscopic objective lens (Obj_1,2,3_: 20 × ) with a numerical aperture of 0.4. L_*n*_ represents lenses with different focal lengths (L_1_ and L_2_: f = 150 mm; L_3_: f = 50 mm). The Obj_1_, L_1_ and pinhole form a spatial filtering and collimation system. The L_1_, L_2_ and iris form a 4f configuration for achieving phase modulation. The length of the MMF is 10 m. **b** The captured speckle pattern by the camera and the estimated speckle pattern evaluated by the calibrated TM, where the Pearson correlation coefficient is used as an evaluation index for the similarity of speckle patterns. The example phase image is a 256-level gray scale image with a resolution of 64 × 64. The resolutions of the speckle patterns are 384 × 384.

The empirical TM of a 10 m MMF can be calibrated using the four-step phase shift method^61^ (See Methods section for details), where the experimental set up is shown in Fig. 2a. As shown in Fig. 2b, the calibrated empirical TM (its size is 147456 × 4096) of a 10 m MMF is used to evaluate the output speckle intensity for a given input light field, where it is compared with the speckle pattern captured by the camera in the real system to evaluate the accuracy of the TM measurement. The predicted speckle pattern is very close to the captured speckle pattern, with correlation about 0.94, which consolidates that the empirical TM is accurate for updating the DENN.

### Simulation and experimental results

**Fig. 3:**
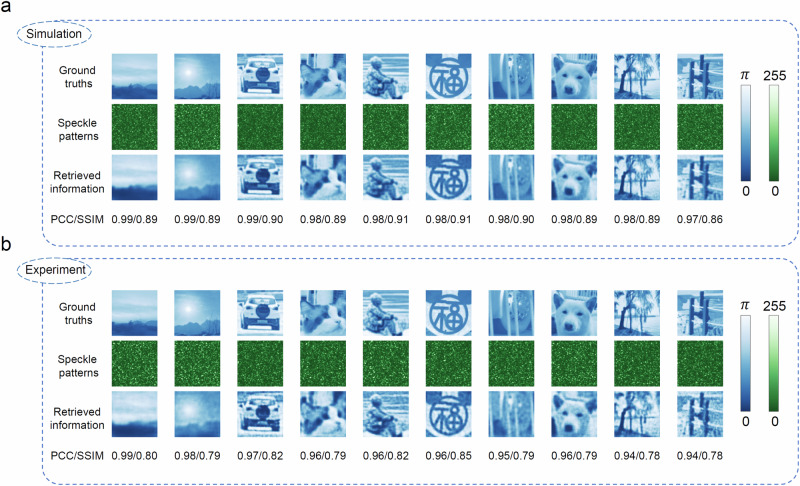
Simulation and experiment retrieval results of phase-encoded natural scene images after passing through a 10 m multimode fiber (MMF). **a** Simulation results using a calibrated TM of the MMF. The ground truths, the estimated output speckle patterns of the MMF and the retrieved information of the DENN are provided, respectively. **b** Experimental retrieval results in a 10 m MMF. The ground truths, the measured output speckle patterns of the MMF and the retrieved information of DENN are provided, respectively. The corresponding structure similarity index measure (SSIM) and Pearson correlation coefficient (PCC) are presented. Colorbars are also provided. These images are all 256-level gray scale images with a resolution of 64 × 64. The resolutions of all speckle patterns are 384 × 384.

The performance of the proposed DENN is studied utilizing the empirical TM in simulation first. The empirical TM of a 10 m MMF is calibrated, which is then used to update the DENN in simulation. Because the phase retrieval is based on the intensity measurement, the phase modulation ranged of the gray scale image is limited in 0-*π* for avoiding multiple solutions. Considering the phase-encoded information of general natural scene images with a resolution of 64 × 64, conventional DNNs are challenging to retrieve the input information over the MMF from limited size of data^63^. On the contrast, the DENN is capable of precisely retrieving the complicated phase-encoded information without any labeled data. The general natural scene images captured by ourselves are similar to those from the ImageNet database^64^, which are complex and sufficient diverse for representing the information in practical applications, as shown in the first row of Fig. 3a. The calibrated empirical TM is used to numerically evaluate the output speckle pattern of the given phase-encoded incident light field, which is shown in the second row of Fig. 3a. The weights and biases of the network are updated to minimize the discrepancy between two speckle patterns. Finally, the corresponding outputs of the DENN are shown in the third row of Fig. 3a, whose Pearson correlation coefficient (PCC)/structure similarity index measure (SSIM) with respect to the ones shown in the first row of Fig. 3a can be up to 0.98/0.91.

**Fig. 4:**
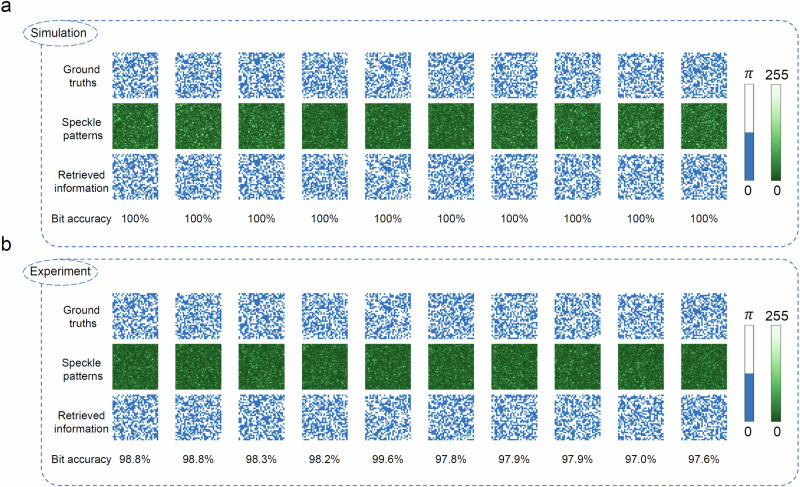
Simulation and experiment retrieval results of phase-encoded uncorrelated binary information after passing through a 10 m MMF. **a** Simulation results using a calibrated TM of the MMF. The ground truths, the estimated output speckle patterns of the MMF and the retrieved binary information of the DENN are provided, respectively. **b** Experimental retrieval results in a 10 m MMF. The ground truths, the measured output speckle patterns of the MMF and the retrieved binary information of the DENN are provided, respectively. The corresponding bit accuracies are given. Colorbars are also provided. The resolution of the binary information is 32 × 32. The resolutions of all speckle patterns are 256 × 256.

These results indicate that the proposed DENN has a good retrieving fidelity when the empirical model is accurate. Experiments are performed in the same MMF to validate the simulation results. As can be seen from the experimental results shown in Fig. 3b, the highest retrieval fidelity (PCC/SSIM:0.96/0.85) is even better than the conventional data-driven supervised learning with tens of thousands of labeled data pairs even if the empirical TM is not with perfect accuracy^63,65^ (see more results in Supplementary Note 2). In order to quantitatively demonstrate the generalization of this method for different types of information, uncorrelated random binary data are also tested in the 10 m MMF under the same conditions, where the simulation and experiment results are shown in Fig. 4a and b, respectively. The achieved averaged bit accuracies are 100% and 98.19% for the simulation and experiment cases, respectively. The slight reductions of fidelity and bit accuracy for the experiment cases compared with the simulation ones shown in Figs. 3 and 4 is because the generated phase wavefront and captured speckle are perfect in simulation. Improving the phase modulation precision of the DMD could reduce this discrepancy.

We further investigate the dependence of performance on the ratio *γ* between the resolutions of speckle pattern and phase-encoded information. Increasing the ratio *γ* can result in a better fidelity evaluated by PCC and SSIM (see Supplementary Note 3). The choice of loss function also has an effect on the fidelity of information retrieval (see Supplementary Note 3). It is found that SSIM_loss_ is a suitable loss function in this case (see Supplementary Note 3) because it can ensure better accuracy of evaluating the similarity between the estimated and measured speckle patterns, which can facilitate the update of weights and biases of the DENN.

### Comparison of performances between a supervised deep neural network and the deep empirical neural network

**Fig. 5:**
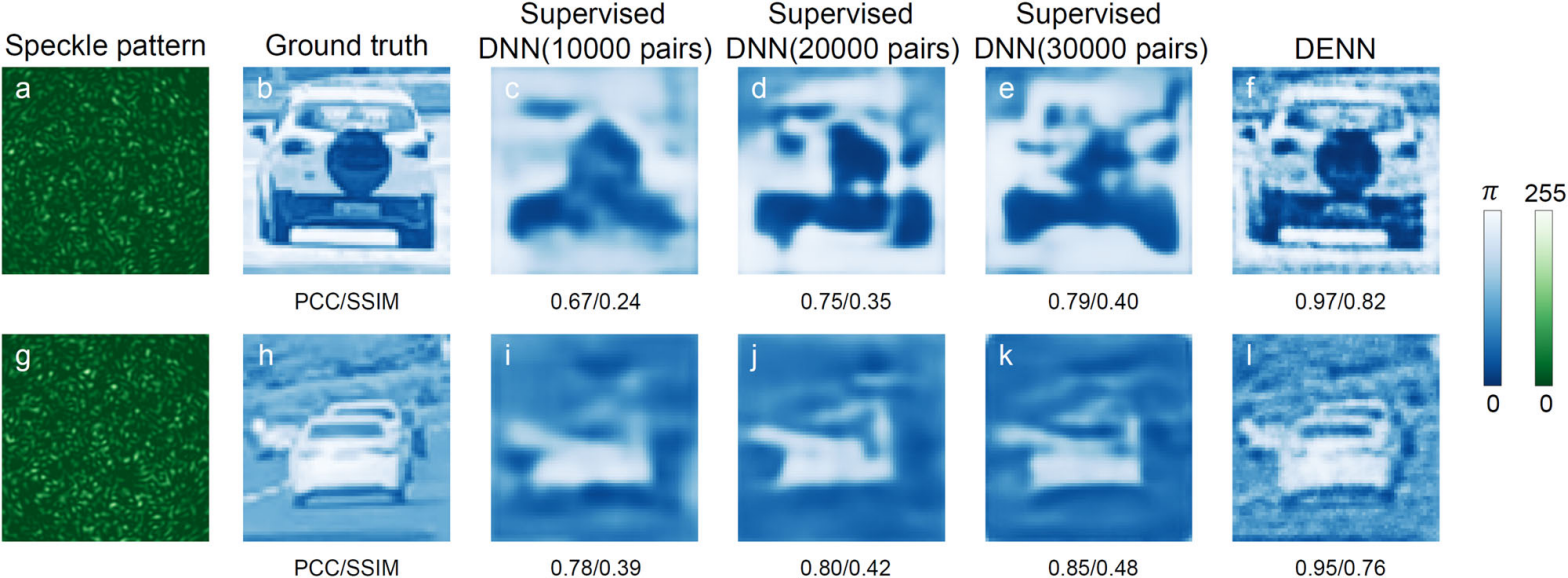
Comparison of performances between a supervised deep neural network (DNN) and the deep empirical neural network (DENN). **a**, **g** The measured output speckle patterns of the 10 m MMF, where the corresponding phase-encoded input information are shown in **b** and **h**, respectively. **c**, **i**, **d**, **j**, **e**, **k** The retrieved results using the supervised DNNs, where their training data amounts are 10,000, 20,000 and 30,000 pairs, respectively. **f**, **l** The retrieved results of DENN. The corresponding structure similarity index measure (SSIM) and Pearson correlation coefficient (PCC) are given. Colorbars are also provided. The resolutions of the natural scene images are 64 × 64. The resolutions of all speckle patterns are 384 × 384.

In order to provide a quantitative comparison with the supervised DNN, the performances of the supervised DNN using different amounts of data are also studied in the same MMF. The supervised DNN employs the same convnet of the DENN. The well-trained supervised DNN can map a speckle pattern to its phase-encoded information with moderate fidelity, where the corresponding training times are approximately 8 hours 34 minutes, 17 hours 13 minutes, and 25 hours 50 minutes using the same training epoch with the DENN for labeled data of 11250, 22500 and 33750 pairs, respectively (see Methods section and Supplementary Note 4 for more details). As shown in Fig. 5, though the fidelity of retrieved phase-encoded information is improved when the data amount of supervised DNN is increased (see Fig. 5c–e and i–k), the retrieved fidelities of two examples using the DENN are much better than the best case of supervised DNNs with different amounts of data, where the SSIM is improved more than 58%. The generalizations of DENN to the scenarios of 1 km MMF (see Supplementary Note 5) and an opaque ground glass (see Supplementary Note 6) showcase the excellent performance of the proposed DENN for optical phase retrieval over different scattering media. Because the calibrated TM contained parasitic noise during measurement, the experimental retrieval results presented above take the noise into account. By artificially adding Gaussian white noise to the estimated speckle and empirical model, respectively, the performance of DENN is shown to be robust to noise (see Supplementary Notes 7 and 8 for the effects of noise on the performance of DENN). The empirical model should be re-calibrated for different scattering media. A higher speed light modulator and a faster camera can be used to further improve the precision of measured empirical TM and reduce the acquisition time, simultaneously. These results further pinpoint the superiority of the DENN leveraged by the empirical TM.

## Discussion

We introduce and demonstrate a concept of DENN for seeing through an opaque medium where the untrained neural network using analytical theory is not applicable. By integrating the convnet with the empirical model that functions as an equivalent FC layer, the phase-encoded information scrambled by MMFs with different lengths and the opaque ground glass can be retrieved in an untrained manner, providing an effective paradigm shift for unscrambling the multiple scattering. The embedding of empirical model to the neural network not only enables the optical phase retrieval over strong scattering media in an untrained manner, but also automatically integrates the real response of the system to facilitate the physical consistency under back propagation of the neural network. General natural scene images that is too diverse to be precisely retrieved using data-driven DNNs can be explicitly retrieved utilizing the DENN, where the highest retrieved fidelity is more than 58% better than the data-driven case (30000 data pairs). The training and inference time of a typical data-driven DNN are about 26 hours and 0.48 second, respectively. While the DENN consumes 270 seconds in inference for a given speckle. Using a more lightweight neural network can reduce the inference time of DENN. The DENN can also be generalized to the homogeneous case, while the methods of diffraction optics^33,34^ cannot be applied in the scattering scenarios.

It should be emphasized that the measurement of empirical TM for a strongly scattering medium cannot be perfect because of residual experimental noises^66^. Though the DENN is quite robust to noise, the performance of the DENN can be improved if the precision of the calibrated TM can be further increased^67,68^. Elements in the TM can become learnable parameters of the DENN, which might further improve the achieved fidelity. We also study the integration of the empirical model to the self-supervised learning model used in homogeneous media^25^ and prDeep^69^ with a pretrained denoiser^70^ (Supplementary Notes 9 and 10), respectively. With the method proposed in prDeep^69^, the fidelity of optical phase retrieval can be further improved using suitable regularization strength (Supplementary Note 10). Future efforts that combine the advantages of self-supervised learning model^25^, prDeep^69^ with a pretrained denoiser^70^, plug-and-play priors^71^ with the DENN can be used to further improve the fidelity of optical phase retrieval over scattering media. Dynamical learning can also be used to improve the robustness to vibration^72^.

By numerically generating TMs with Gaussian distributions that can mimic the physical properties of various strongly scattering media^73^, it is shown that the DENN can retrieve general natural scene images and uncorrelated binary information with high accuracy over different scattering media (see Supplementary Note 11 for more details). These results indicate that the proposed DENN is general and can be applied to various scattering media whose empirical TM can be measured. It can enable the high fidelity optical phase retrieval with only a single-shot intensity measurement, providing a potential solution for the general but long standing challenging in optical communication, optical metrology, and holography, where various transmissive channels suffer from unwanted scattering. The nonlinear effects of the multiple scattering media are not considered yet^74,75^ in this work, which could inspire new applications. There are still limitations of the proposed DENN. The inference time and the size of speckle pattern required can be further reduced. It is anticipated that the DENN can not only provide new insight for the black box problem of strongly scattering media, but also shed new light on much broader disciplines of unknown wave scattering systems relied on the data-driven DNNs^76,77^.

## Methods

### Experimental setup

The optical setup for calibrating the empirical TM of the MMF is shown in Fig. 2a, where a CW laser (wavelength is 532 nm, MSL-S-532-50mW CH80136, CNI) is collimated and expanded through the combination of an objective lens (Obj_1_), a pinhole, and a lens (L_1_). The collimated beam is modulated by a DMD (F4320 DDR 0.95 1080P, Fldiscovery), where the first-order diffractive beam^78^ is selected using a 4f filtering system composed of a lens (L_2_), an iris and a lens (L_3_). Then the laser beam with modulated phase is coupled to the proximal end of a 10 m step refractive index MMF (diameter is 105 *μ*m, NA = 0.22, YOFC) through an objective lens (Obj_2_). The scattered light field at the distal end of the MMF is collected by another objective lens (Obj_3_), where the speckle pattern is recorded by a charge coupled device (CCD) camera (Hypersen). A linear polarizer in front of the camera is used to ensure the horizontal polarization of the captured speckle pattern. The four-step phase shift method is used to calibrate the empirical TM^61^.

### Data acquisition and preprocessing

For the DENN, the resolutions of the speckle patterns input to the network are 384 × 384 and 256 × 256 for the general natural scene images and uncorrelated binary information, respectively. The output for binary information by the DENN is binarized. The uncorrelated binary information is generated by computer with uniform random distribution. The speckle patterns for 100 phase-encoded natural scene images (64 × 64 pixels) and 100 phase-encoded binary information (32 × 32 pixels) are taken to validate the DENN.

For the supervised DNN, the acquired labeled data (50000 pairs with speckle patterns and phase-encoded information) is randomly sampled to form datasets of 12500, 25000 and 37500 pairs for the supervised learning, respectively, where the phase-encoded information is a combination of images from the ImageNet dataset^64^ and natural scene images captured by ourselves. Each dataset is divided into a training set (80%), a validation set (10%), and a test set (10%). In order to provide a fair comparison of performance between the DENN and the supervised DNN, the resolution of the speckle patterns input to the supervised DNN is the same as the one of DENN.

The natural scene images shown in all figures are captured by ourselves.

### Update configuration of the neural network

Both the DENN and the supervised DNN are implemented in PyTorch 1.10.2 using the python version 3.6 platform, which are carried out on a desktop with an i5-12400F CPU and an NVIDIA RTX 3090 GPU. The optimizer adopted is a newer version of Adam, with an initial learning rate of 4 × 10^−4^ for AdamW^60^, optimized every 5 times, rising to 0.001 after the first 5 training epochs, and then gradually dropping to 0 as the rounds increase in order to optimize the weights and biases of the network^79^. The epochs trained by the DENN and supervised DNN are both 120. The loss function is SSIM_loss_ (see Supplementary Note 12) while the definitions of evaluation indicators for phase retrieval are presented in Supplementary Note 13.

## Supplementary information

Supplementary Information

Transparent Peer Review file

## Data Availability

The example data generated in this study for the DENN and supervised DNN are available at: https://zenodo.org/records/10938116. Any additional data are available from Yi Xu (yixu@ gdut.edu.cn) upon request. Source data are provided with this paper.

## Code availability

The Python codes used in this paper are available at https://zenodo.org/records/10938116.
