# Supplementary material for: Deep empirical neural network for optical phase retrieval over a scattering medium
Source: Nat Commun. 2025 Feb 5;16:1369. doi: 10.1038/s41467-025-56522-5 (PMC11799312; doi:10.1038/s41467-025-56522-5)
Supplement: Supplementary file 1 — Supplementary Information [file 41467_2025_56522_MOESM1_ESM.pdf]

Supplementary Information for Deep empirical neural network for optical phase retrieval over a scattering medium

Huaisheng Tu[1,2,3,4,†], **Haotian Liu**[1,2,3,4,†], **Tuqiang Pan**[1,2,3,4], **Wuping Xie**[1,2,3,4], **Zihao Ma**[1,2,3,4], **Fan Zhang**[1,2,3,4], **Pengbai Xu**[1,2,3,4], **Leiming Wu**[1,2,3,4], **Ou Xu**[1,2,3,4], **Yi Xu**[1,2,3,4,*], **and Yuwen Qin**[1,2,3,4,*]

[1]Key Laboratory of Photonic Technology for Integrated Sensing and Communication, Ministry of Education, Guangdong University of Technology, Guangzhou, 510006, China
[2]Guangdong Provincial Key Laboratory of Information Photonics Technology, Guangdong University of Technology, Guangzhou, 510006, China
[3]School of Information Engineering, Guangdong University of Technology, Guangzhou, 510006, China
[4]Institute of Advanced Photonic Technology, Guangdong University of Technology, Guangzhou, 510006, China
[†]These authors contributed equally: Huaisheng Tu, Haotian Liu
[*]Corresponding authors: yixu@gdut.edu.cn, qinyw@gdut.edu.cn

Supplementary Note 1 — Architecture of U^{2}Net

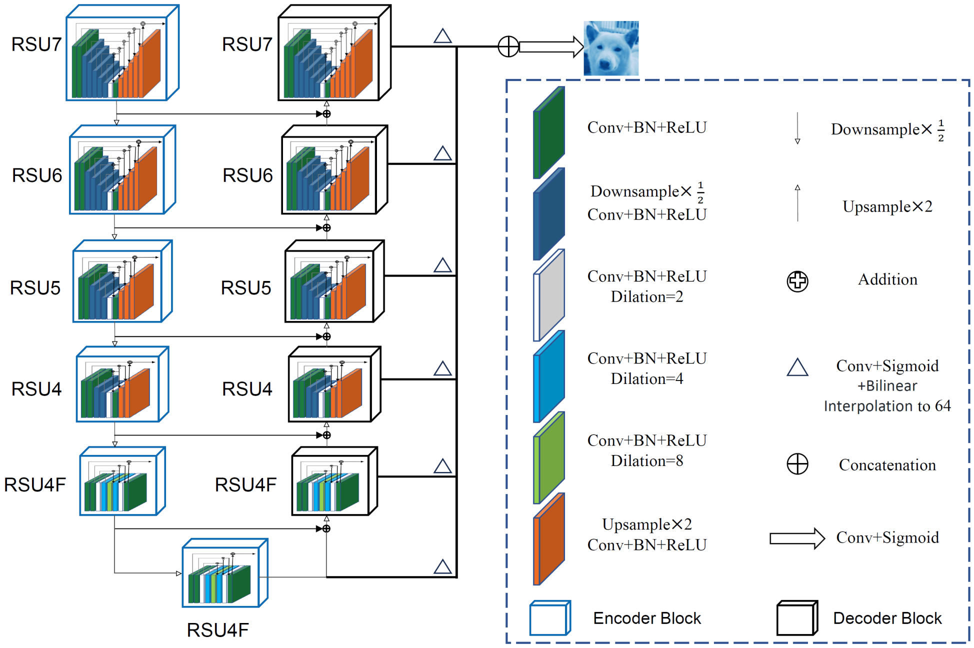

Supplementary Figure 1. Architecture of U^{2}Net. The U^{2}Net takes a general encoder–decoder Unet structure. With two level nested U-structures, U^{2}Net can capture more local and global information from both deep and shallow layers. Here, RSU is a Residual U-block, Conv is a convolutional layer, BN indicates batch normalization, ReLu is a rectified linear unit. The typical natural scene image is captured by ourselves.

As shown in Supplementary Figure 1, the deep neural network (DNN) for integrating the empirical TM is a U^{2}Net with a lightweight version[1], whose parameters are 1/40 of the full type U^{2}Net. It consists of six encoder blocks, five decoder blocks, and a map fusion module. All blocks are designated as Residual U-blocks (RSUs),

which replace the convolutional layers of the original Unet architecture. The RSU is a modified residual block consisted of three distinct components. The first part is the input convolution layer, which converts the input feature map into an intermediate feature map with a specified channel number for local feature extraction. The next part is a Unet-like symmetric encoder-decoder structure with a depth of L and an intermediate feature map input for learning multi-scale context information. For example, RSUL means that it consists of L encoding layers and L-1 decoding layers, where skip connections are used to preserve high frequency information. The encoder layer comprises a convolutional block followed by a down-sampling layer, whereas the decoder layer comprises a convolutional block followed by an up-sampling layer. Each convolutional block is composed of a convolutional layer with a kernel size of 3×3, batch normalization (BN), and rectified linear unit (ReLU) activation. In particular, RSU4F indicates that the down-sampling and up-sampling operations within RSU4 are replaced by dilated convolutions. This is because the length and width of the feature map are small at this stage, and further down-sampling may lead to loss of context information. The last part is the fusion of local features and multi-scale features through residual connection, which helps to retain the information of the original features and improve the expression ability of the network. In the map fusion module, all feature maps from the decoders and RSU4F at the bottom of Supplementary Figure 1 pass through a convolutional layer with a convolutional kernel size of 3×3, each of which results in an output channel. Then the corresponding output is scaled to the specified size using bilinear interpolation, which are concatenated among channels to form a feature map with channel number of 6. Finally, the feature map passes through a convolution layer with a kernel size of 1×1 and the output channels number is 1. The Sigmoid function is used to limit their value range from 0 to 1 as the final prediction result of the network. Due to the repetitive Unet structure in the U^2Net, the adopted U^2Net allows for simultaneous extraction of local and global information on each RSU. This feature makes the U^2Net suitable for handling nonlocal effects in strong scattering media.

Supplementary Note 2 — The phase retrieval results through a 10 m MMF using the other type of gray scale images

Simulation and experimental results of phase-encoded human face images transmission over a 10 m long MMF are provided to further validate the generality of the proposed deep empirical neural network (DENN). The fidelity of phase retrieval can be evaluated by Pearson correlation coefficient (PCC) and structure similarity index measure (SSIM). The ground truths, estimated speckle patterns, and retrieved information are shown in the first, second and third rows of Supplementary Figure 2a, respectively, where the achieved averaged retrieval fidelity is 0.989/0.879 (PCC/SSIM). Accordingly, the experimental results are shown in Supplementary Figure 2b, where the input of the network comes from the speckle pattern captured by the camera. The achieved averaged PCC/SSIM is 0.974/0.769, which is a little bit smaller than the simulation case.

Supplementary Note 3 — The retrieval performances using different γ and loss functions

The dependences of phase retrieval performance of the DENN on the γ that is the ratio between the resolutions of the speckle pattern and the phase-encoded information are shown in Supplementary Figure 3a. Four different resolutions of speckle patterns are presented, i.e. $64 \times 64(\gamma = 1)$, $128 \times 128(\gamma = 4)$, $256 \times 256(\gamma = 16)$ and $384 \times 384(\gamma = 36)$, respectively. The phase-encoded wavefront, TM and speckle intensity for evaluating the loss function in simulation are perfect in principle. While there is parasitic noise in generated phase-encoded wavefront, calibrated TM and captured speckle, which results in the discrepancy between simulation and experiment. As can be seen from this figure, increasing γ can dramatically enhance the performance of the DENN, where the retrieval fidelity can be improved by selecting a suitable and available ratio γ.

The influences of using mean absolute error (MAE), mean squared error (MSE) and $SSIM_{loss}$ (see Supplementary Note 12 for their definitions) as the loss function of the DENN are shown in Supplementary Figure 3b. Here, the resolution of the phase-encoded image is 64×64 and the resolution of speckle pattern is 384×384. It can be found that the case of using $SSIM_{loss}$ as a loss function possesses the best performance, because the $SSIM_{loss}$ can not only consider pixel-level differences but also focus on the nonlocal properties of the information.

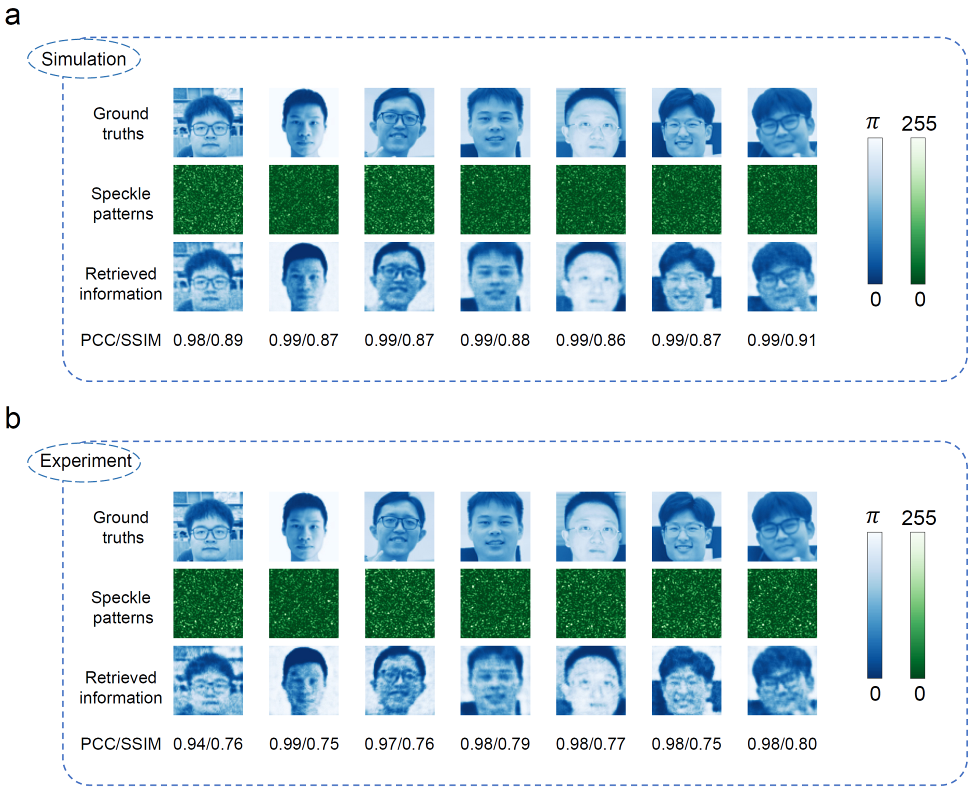

Supplementary Figure 2. Simulation and experiment retrieval results of phase-encoded human face images after passing through a 10 m MMF. a Simulation results using an empirical TM of an MMF. The ground truths, the estimated output speckles of the MMF and the retrieved phase-encoded information of the DENN are provided. **b** Experimental retrieval results through a 10 m MMF. The ground truths, the measured output speckle patterns of the MMF and the retrieved information of the DENN are provided, respectively. The corresponding structure similarity index measure (SSIM) and Pearson correlation coefficient (PCC) are presented. Colorbars are also provided. These human face images are students from Guangdong University of Technology with their permissions for use, where they are all 256-level gray scale images with a resolution of 64×64. While the resolutions of all speckle patterns are 384×384.

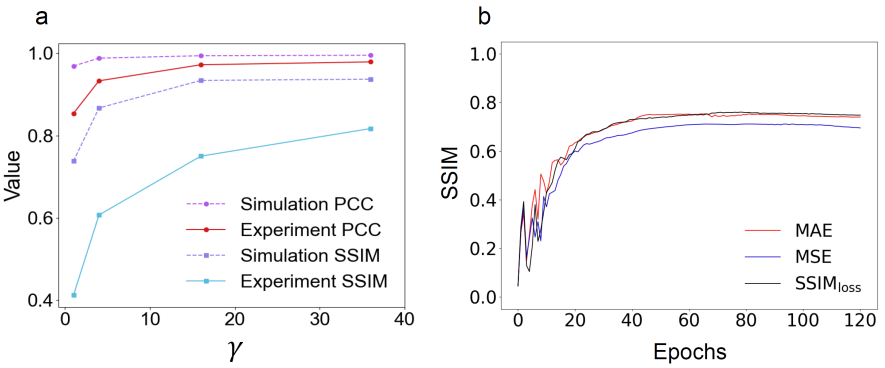

Supplementary Figure 3. The influences of ratio γ and loss functions on the performances of the deep empirical neural network. a The dependences of retrieval performances on the ratio (γ) between the resolutions of the speckle pattern and the phase-encoded information for a 10 m long MMF. Both simulation and experimental results of Pearson correlation coefficient (PCC) and structure similarity index measure (SSIM) are presented. **b** The effects of different loss functions on the retrieval performances for a 10 m long MMF. Here, MAE, MSE and $SSIM_{loss}$ represent the mean absolute error, mean squared error and structure similarity index measure error, respectively.

Supplementary Note 4 — Comparison between the traditional supervised deep neural network and the deep empirical neural network

Comparison items	Supervised deep neural network			Deep empirical neural network
Empirical model	N/A			**Empirical TM**
Data type	Natural scene images			
Data amount	Train: 10000 pairs<br>Validation 1250 pairs<br>Test: 1250 pairs	Train: 20000 pairs<br>Validation: 2500 pairs<br>Test: 2500 pairs	Train: 30000 pairs<br>Validation: 3750 pairs<br>Test: 3750 pairs	**1 speckle pattern**
Computational time for 1 speckle pattern	Train: $\sim$ 8 hours 34 minutes<br>Inference: $\sim$ 0.48 second	Train: $\sim$ 17 hours 13 minutes<br>Inference: $\sim$ 0.48 second	Train: $\sim$ 25 hours 50 minutes<br>Inference: $\sim$ 0.48 second	**Inference: $\sim$ 4 minutes 30 seconds**
Averaged PCC/SSIM	0.798/0.453	0.827/0.502	0.836/0.527	**0.951/0.689**

Supplementary Table 1. Comparison between the traditional supervised deep neural network and the deep empirical neural network (DENN). The corresponding structure similarity index measure (SSIM) and Pearson correlation coefficient (PCC) are presented, where the PCC/SSIM of DENN is averaged over 100 realizations. N/A indicates not applicable and TM indicate transmission matrix.

Supplementary Note 5 — Comparison of performances between the speckle-correlation scattering matrix and the deep empirical neural network

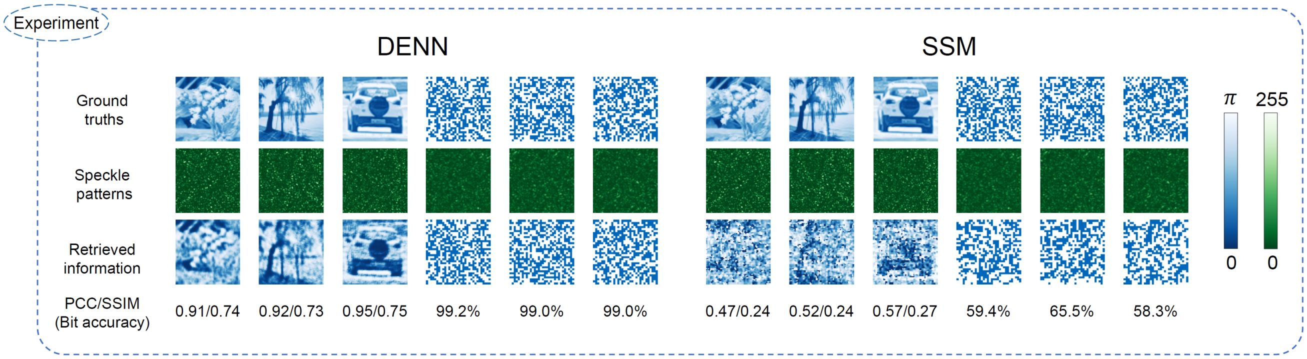

Supplementary Figure 4. Comparison of performances between the speckle-correlation scattering matrix (SSM) method and the deep empirical neural network (DENN) over a 1 km MMF. The ground truths, the measured output speckle patterns and the retrieved information of DENN and SSM are provided, respectively. The resolutions of the natural scene images captured by ourselves are 64×64. While the resolutions of the speckle patterns are 384×384. The resolutions of the uncorrelated binary information are 32×32. While the resolutions of the speckle patterns are 256×256. The corresponding structure similarity index measure (SSIM), Pearson correlation coefficient (PCC) and bit accuracy are presented. Colorbars are also provided.

We perform quantitative comparison between the single-shot optical phase retrieval using the state-of-the-art method of speckle-correlation scattering matrix (SSM)[2] and the DENN over a much longer MMF (1 km, step index, diameter is 200 μm, NA = 0.22, YOFC). The SSM method is also based on the empirical TM, where a sufficiently large ratio (γ) between resolutions of the speckle pattern and the encoded information is necessary for achieving high fidelity optical phase retrieval. It relies on the approximation that the sole eigenvector of the scattering matrix is the retrieval incident field under a sufficiently large γ. As shown in Supplementary Figure 4, the retrieval results of

the SSM method are not as good as the 1 km MMF by using a near infrared laser(wavelength is 850 nm, Toptica). Because the empirical TM does not rely on approximation, the retrieved PCC/SSIM of natural scene images using the DENN is as high as 0.95/0.75 and the retrieved accuracy of binary information using the DENN is as high as 99.2% under the same experimental conditions, showing good superiority of the DENN for optical phase retrieval over a scattering medium.

Supplementary Note 6 — The experimental results over an opaque ground glass

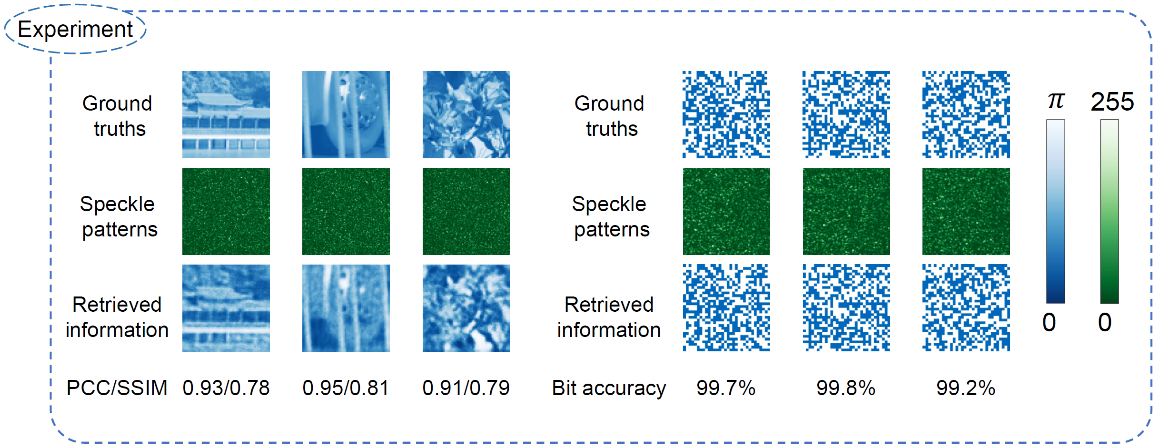

Supplementary Figure 5. Experiment retrieval results of phase encoded natural scene images and uncorrelated binary information over an opaque ground glass. The ground truths, the measured output speckle patterns and the retrieved information of DENN are provided, respectively. The natural scene images captured by ourselves are all 256-level gray scale images with a resolution of 64×64. The corresponding resolutions of speckle patterns are 384×384. The resolutions of the binary information are 32×32 and the resolutions of speckle patterns are 256×256. The corresponding structure similarity index measure (SSIM), Pearson correlation coefficient (PCC) and bit accuracy are presented. Colorbars are also provided.

To demonstrate the generality of DENN, we study the optical phase retrieval over the other scattering medium, i.e. an opaque ground glass (DG10-1500, Thorlabs), where the experimental results are shown in the Supplementary Figure 5. The retrieved PCC/SSIM of natural scene images using the DENN is as high as 0.95/0.81 and the retrieved accuracy of binary information is as high as 99.8%, showing good generality of the DENN for achieving optical phase retrieval over different scattering media.

Supplementary Note 7 — The influences of speckle accuracy on the performances of deep empirical neural network

As shown in Supplementary Figure 6, four scenarios of speckle accuracy (cor. = 0.5, 0.75, 0.9, 1) are studied by adding different amounts of Gaussian white noise to the estimated speckle pattern. It can be seen that when cor. $\geq$ 0.75, the achieved PCC/SSIM is as high as 0.96/0.82. The achieved PCC/SSIM is still higher than 0.94/0.74 when cor. = 0.5.

Supplementary Note 8 — The influence of noise in the empirical TM on the performance of deep empirical neural network

We quantitatively evaluate the performance of DENN when the empirical TM is perturbed by different amounts of noise in simulation. Different amounts of Gaussian white noise are added to the empirical TM, where the corresponding signal to noise ratio (SNR) varies between 5 and 100 (SNR = 5, 10, 20, 40, 60, 80, 100). From

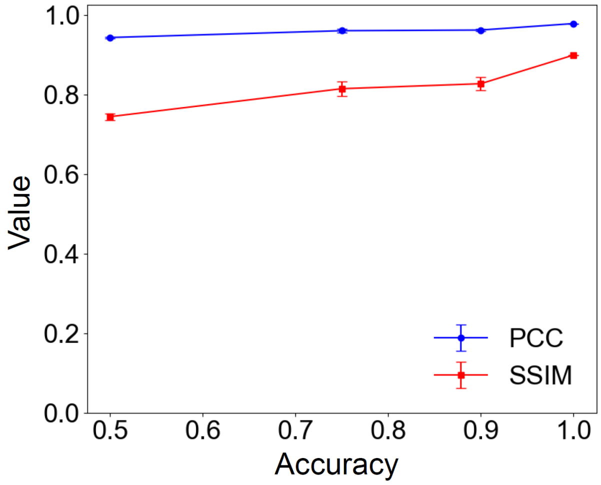

Supplementary Figure 6. The dependences of retrieval fidelity on the accuracy of estimated speckle intensity calculated by simulation. Here, the speckle accuracy is measured by Pearson correlation coefficient (PCC). The noise added in the speckle is Gaussian white noise. The resolution of the phase-encoded information is 64×64 and the corresponding resolutions of speckle patters are 384×384. The TM is calibrated in a 1 m long MMF. The retrieval PCC and structure similarity index measure (SSIM) are presented. The error bars for 6 realizations are shown, which indicate the corresponding standard deviations.

the results shown in Supplementary Figure 7, it can be seen that the phase retrieval fidelity of DENN is robust to different levels of noise.

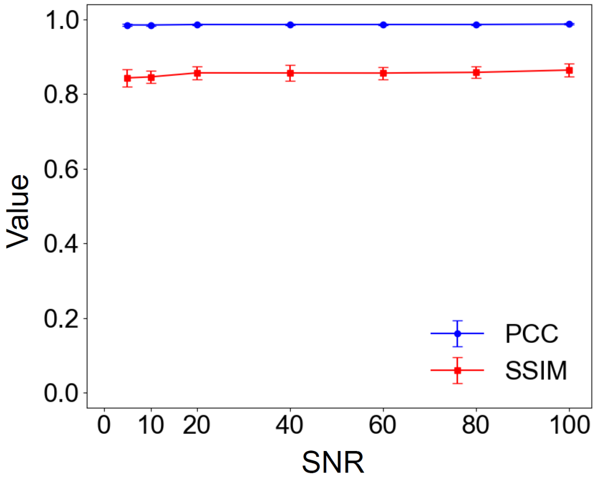

Supplementary Figure 7. The dependences of retrieval fidelity on adding different signal-to-noise ratios (SNRs) to the empirical TM of DENN calculated by simulation. The resolutions of the phase-encoded information are 64×64 and the corresponding resolutions of speckle patterns are 384×384. The TM is calibrated in a 1m long MMF. The retrieval structure similarity index measure (SSIM) and Pearson correlation coefficient (PCC) are presented. The error bars for 6 realizations are shown, which indicate the corresponding standard deviations.

Supplementary Note 9 — The performances of the self-supervised learning method for optical phase retrieval over a scattering medium

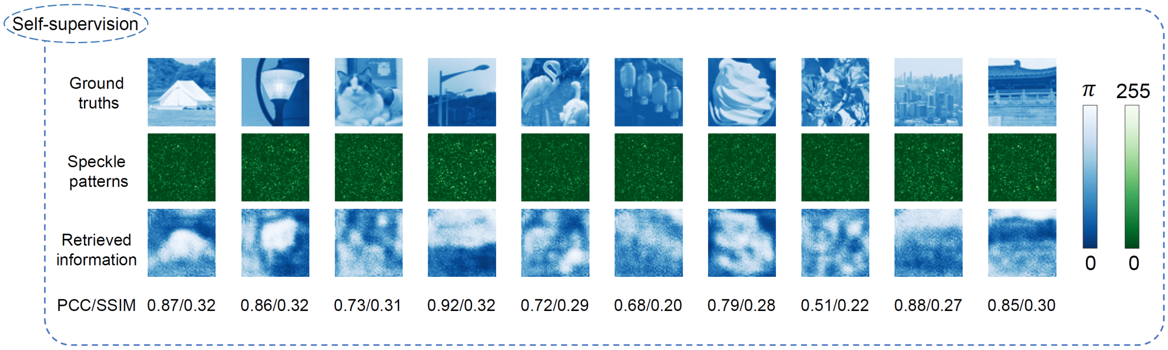

Supplementary Figure 8. Performance of optical phase retrieval over a 10 m MMF using the self-supervised learning method. The ground truths, the measured output speckle patterns and the retrieved information of DENN are provided, respectively. The natural scene images captured by ourselves are 256-level grayscale images with a resolution of 64×64. Their corresponding resolutions of the speckle patterns are 384×384. The corresponding structure similarity index measure (SSIM) and Pearson correlation coefficient (PCC) for the natural scene images are indicated. Colorbars are also provided.

A self-supervised learning scheme[3] used in homogeneous media is adapted to deal with the problem of optical phase retrieval over a scattering medium. A TM calibrated for a 10 m MMF is used as the empirical model. 12500 unlabeled speckle patterns are used where they are divided into training set, validation set and test set in a ratio of 8:1:1. $\text{SSIM}_{\text{loss}}$ and AdamW are still used as loss functions and optimizers for the self-supervised network, respectively. After 40 epochs and 78 hours of training, the retrieved average PCC/SSIM of natural scene images of the test set is 0.783/0.294, as shown in Supplementary Figure 8.

Supplementary Note 10 — Performances of deep empirical neural network with and without regularization by denoising utilizing a pretrained denoiser

We also quantitatively study performances of DENN with and without regularization by denoising utilizing a pretrained denoiser[4] in two experiments. First, we generate the training dataset by adding Gaussian white Noise (std = 30 dB) to 10,000 images from ImageNet[5]. We get the denoiser by utilizing DnCNN[6] to fit the training dataset. The $\text{SSIM}_{\text{loss}}$ and AdamW are still used as the loss functions and optimizer to minimize the following expressions:

$$\varphi^*(x,y) = \underset{\widetilde{\varphi},m}{\arg\min} \left\| |Te^{i\cdot F_m(I)\cdot\pi}|^2 - I \right\|^2 + \frac{\lambda}{2}\widetilde{\varphi}^T\left(\widetilde{\varphi} - R_{m^*}(\widetilde{\varphi})\right) \tag{1}$$

where F_m is our neural network mapping the speckle intensity I to the phase $\widetilde{\varphi}$, λ is the RED regularization strength, and R_{m^*} is the pre-trained denoising model. The corresponding comparison are shown in Supplementary Figure 9. The performance of DENN can be improved using suitable regularization strength (λ).

Supplementary Note 11 — The phase retrieval results using randomly generated empirical models

In order to further showcase the generality of the DENN, we numerically study the scenario of empirical TM whose elements are randomly diffused with a Gaussian distribution. This kind of TM can simulate the empirical TMs of various diffusers regardless of thickness, scattering strength, and TM size if the column vectors of the TM are orthogonal[2]. As shown in Supplementary Figure 10a and b, the highest PCC/SSIM achieved for the retrieved natural

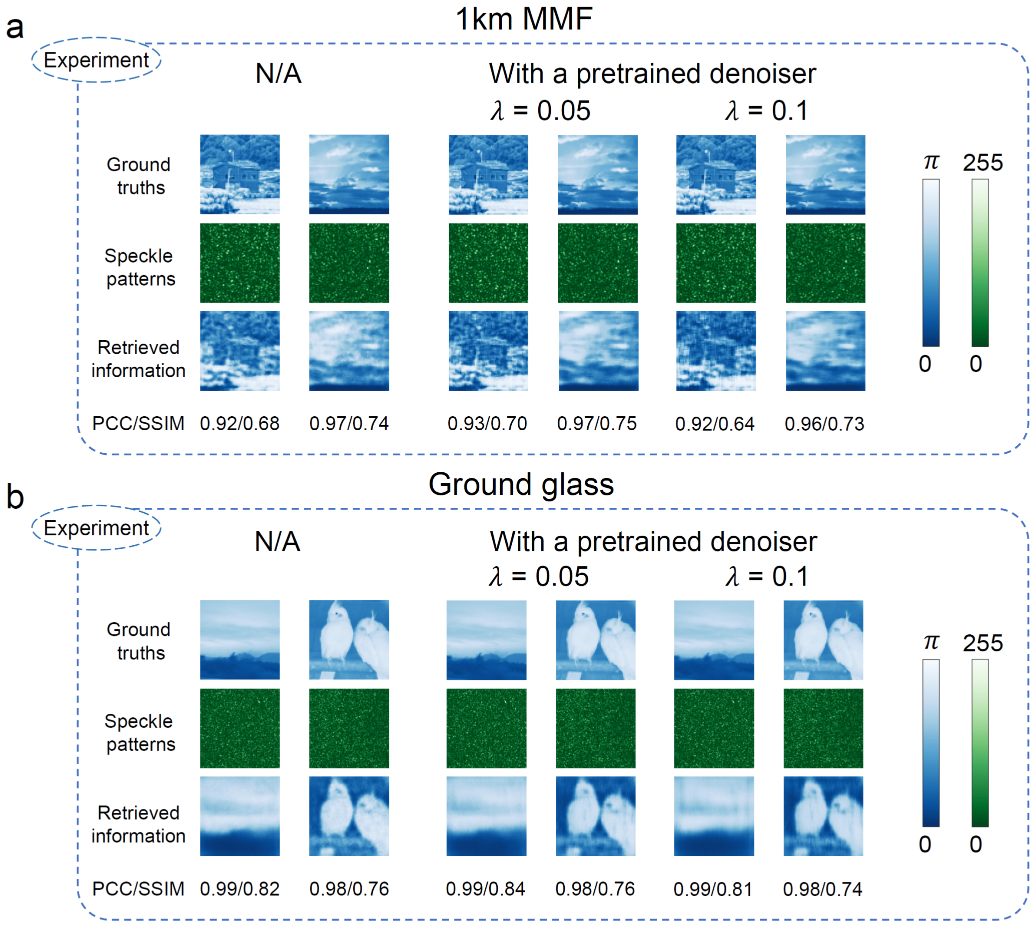

Supplementary Figure 9. Performances of deep empirical neural network (DENN) with and without regularization by denoising (RED) utilizing a pretrained denoiser in two experiments: a 1 km multimode fiber(MMF) (a) and a ground glass (b). The ground truths, the captured output speckle patterns of the MMF/ground glass and the retrieved information of DENN with and without regularization by denoising utilizing a pretrained denoiser are provided, respectively. Here, two cases (λ = 0.05, 0.1) are provided. These natural scene images captured by ourselves are all 256-level gray scale images with a resolution of 64×64. Their corresponding resolutions of the speckle patterns are 384×384. The corresponding structure similarity index measure (SSIM) and Pearson correlation coefficient (PCC) are presented. Colorbars are also provided. N/A indicates not applicable.

scene images is 0.99/0.95 and the bit accuracies for the retrieved uncorrelated binary information are all 100%, respectively. These results further consolidate the generality and superiority of the DENN.

Supplementary Note 12 — Definition of three kinds of loss functions

The loss function of structure similarity index measure is used as the first kind of loss function to train the network, where is given as

$$\mathrm{SSIM}_{\mathrm{loss}} = 1 - \frac{(2\mu_Y\mu_G + c_1)(2\sigma_{YG} + c_2)}{(\mu_Y^2 + \mu_G^2 + c_1)(\sigma_Y^2 + \sigma_G^2 + c_2)} \tag{2}$$

where Y and G represent the input speckle and estimated speckle of the DENN, respectively. μ_Y and μ_G are the local means of Y and G, respectively. σ_Y^2 and σ_G^2 are the variances of Y and G, respectively. σ_{YG} is the cross covariance of Y and G. And c_1 and c_2 are regularized parameters.

a

b

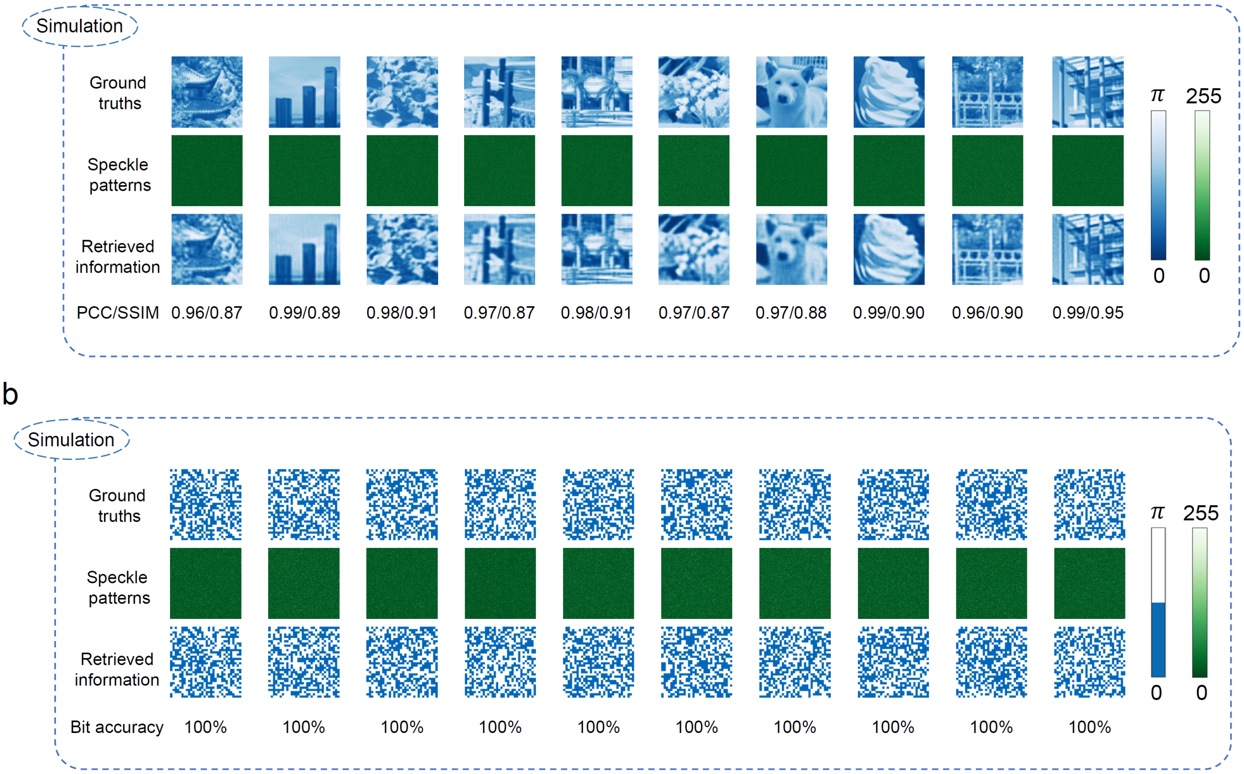

Supplementary Figure 10. Simulation retrieval results of phase-encoded natural scene images and uncorrelated binary information using randomly generated empirical transmission matrix with Gaussian distributions. a The ground truths, the estimated output speckles under randomly generated empirical TM and the retrieved information are provided. The resolutions of the natural scene images captured by ourselves are 64×64. While the resolutions of the speckle patterns are 384×384. **b** The ground truths, the estimated output speckles under randomly generated empirical TM and the retrieved binary information are provided. The uncorrelated binary information is generated by computer with uniform random distribution. The resolutions of the uncorrelated binary information are 32×32. While the resolutions of the speckle patterns are 256×256. The corresponding structure similarity index measure (SSIM), Pearson correlation coefficient (PCC) and bit accuracies are presented. Colorbars are also provided.

At the same time, MAE can be defined as

$$\mathrm{MAE} = \frac{1}{wh}\sum_{i=1}^{w}\sum_{j=1}^{h}|y(i,j) - \hat{y}(i,j)| \tag{3}$$

where w and h specify the resolutions of the images, respectively. y and $\hat{y}$ represent the input speckle and estimated speckle of the DENN, respectively.

Finally, MSE is defined as follows :

$$\mathrm{MSE} = \frac{1}{wh}\sum_{i=1}^{w}\sum_{j=1}^{h}|y(i,j) - \hat{y}(i,j)|^2 \tag{4}$$

where w and h are the resolutions of the images, respectively. y and $\hat{y}$ represent the input speckle and estimated speckle of the DENN, respectively.

Supplementary Note 13 — Evaluation indicators for the retrieved information

The quantitative fidelity of the retrieved gray scale image can be evaluated using the PCC and SSIM. The definition of PCC is as follows:

$$\mathrm{PCC} = \frac{\sum_{i=1}^{w}\sum_{j=1}^{h}(Y(i,j)-\overline{Y})(G(i,j)-\overline{G})}{\sqrt{\sum_{i=1}^{w}\sum_{j=1}^{h}(Y(i,j)-\overline{Y})^2}\sqrt{\sum_{i=1}^{w}\sum_{j=1}^{h}(G(i,j)-\overline{G})^2}} \tag{5}$$

where w and h are the resolutions of the images. $Y(i,j)$ and $G(i,j)$ represent the values of the Y and G under evaluation in row i and column j, respectively. And $\overline{Y}$ and $\overline{G}$ are the averaged values of Y and G, respectively.

While SSIM is defined as

$$\mathrm{SSIM} = \frac{(2\mu_Y\mu_G+c_1)(2\sigma_{YG}+c_2)}{\left(\mu_Y^2+\mu_G^2+c_1\right)\left(\sigma_Y^2+\sigma_G^2+c_2\right)} \tag{6}$$

where Y and G represent two images under evaluation, respectively. μ_Y and μ_G are the local means of Y and G, respectively. σ_Y^2 and σ_G^2 are the variances of Y and G, respectively. σ_{YG} is the cross covariance of Y and G. And c_1 and c_2 are regularized parameters.

Bit accuracy is used as an evaluation indicator for evaluating the fidelity of the retrieved binary information, which is defined as follows:

$$\mathrm{Accuracy} = \frac{1}{wh}\sum_{i=1}^{w}\sum_{j=1}^{h}A(y,\hat{y}), \quad A(y,\hat{y}) = \begin{cases} 1 & y(i,j)=\hat{y}(i,j) \\ 0 & y(i,j)\neq\hat{y}(i,j) \end{cases} \tag{7}$$

where w and h are the resolutions of the images. $A(y,\hat{y})$ is a logical function. y and $\hat{y}$ are the ground truth and the retrieved result of the DENN, respectively.

References

1. Qin, X. *et al.* U2-net: Going deeper with nested u-structure for salient object detection. *Pattern Recognit.* **106**, 107404 (2020).
2. Lee, K. & Park, Y. Exploiting the speckle-correlation scattering matrix for a compact reference-free holographic image sensor. *Nat. Commun.* **7**, 13359 (2016).
3. Huang, L., Chen, H., Liu, T. & Ozcan, A. Self-supervised learning of hologram reconstruction using physics consistency. *Nat. Mach. Intell.* **5**, 895–907 (2023).
4. Metzler, C., Schniter, P., Veeraraghavan, A. & Baraniuk, R. prdeep: Robust phase retrieval with a flexible deep network. In *International Conference on Machine Learning*, 3501–3510 (PMLR, 2018).
5. Deng, J. *et al.* Imagenet: A large-scale hierarchical image database. In *2009 IEEE Conference on Computer Vision and Pattern Recognition*, 248–255 (2009).
6. Zhang, K., Zuo, W., Chen, Y., Meng, D. & Zhang, L. Beyond a gaussian denoiser: Residual learning of deep cnn for image denoising. *IEEE Trans. Image Process.* **26**, 3142–3155 (2017).
