# Supplementary material for: Deep empirical neural network for optical phase retrieval over a scattering medium
Source: Nat Commun. 2025 Feb 5;16:1369. doi: 10.1038/s41467-025-56522-5 (PMC11799312; doi:10.1038/s41467-025-56522-5)
Supplement: Supplementary file 2 — Transparent Peer Review file [file 41467_2025_56522_MOESM2_ESM.pdf]

nature portfolio

Peer Review File

Deep empirical neural network for optical phase retrieval over a scattering medium

Corresponding Author: Professor Yi Xu

This manuscript has been previously reviewed at another journal. This document only contains information relating to versions considered at Nature Communications.

Parts of this Peer Review File have been redacted as indicated to remove third-party material.

This file contains all reviewer reports in order by version, followed by all author rebuttals in order by version.

A version of this paper was originally rejected for publication by Nature Communications, however that decision was reconsidered after appeal by the authors.

Version 0:

Reviewer comments:

Reviewer #1

(Remarks to the Author)
The manuscript presents a method that combines Deep Image Prior (DIP) and the four-step phase-shifting method to address the inverse problem in a Multiple-Input Multiple-Output (MIMO) scattering system. The topic of work is interesting, and the authors provide a new perspective on addressing the inverse problem in MIMO scattering systems. However, I cannot recommend the manuscript to be published with current form. Following major comments should be addressed well.
1. Clarification of Innovation:
The proposed method, while integrating two existing techniques, does not appear to fully explain its innovative aspects. The authors are advised to discuss in more detail the novelty of DENN compared to existing technologies, especially the theoretical or methodological innovations made in the process of combining DIP and the four-step phase-shifting method.
2. Comparison of Data and Time:
The paper claims a reduction in the amount of training data and time, but DENN is essentially an unsupervised learning method. Comparing it with traditional supervised learning DNNs may require more consideration. The authors are encouraged to provide comparisons with traditional unsupervised or self-supervised learning methods to more accurately demonstrate the advantages of DENN.
3. Accuracy of Time Measurement:
When discussing performance improvements, comparing the iterative inference time of the network with training time may not be appropriate. The authors should clarify the distinction between training time and inference time and use accurate terminology in the discussion to avoid confusion.
4. Adequacy of Comparative Experiments:
The paper requires more comparative experiments to demonstrate the superiority of the proposed method. The authors are advised to consider comparing with other existing technologies or methods, especially in terms of performance under different conditions and the application effects on different types of scattering media or inverse problems.
5. Comparison with Related Work:
It is suggested that the authors provide a more comprehensive discussion of related work in the literature review, especially those studies that are similar to or related to the DENN method.
6. Strengthening of the Conclusion:
The conclusion section should provide a more comprehensive summary of the main contributions of DENN and discuss its potential for future research and practical applications.

(Remarks on code availability)

Reviewer #2

(Remarks to the Author)
------------- Summary -----------

Problem, Motivation & Goal: This work considers the problem of retrieving signals from scattering measurements. Specifically, the considered signal is a 2D image (phase) and the measurements are the intensity of the light after transmitting a multi-mode fiber (MMF). Deep learning (DL) is a desired tool for solving the problem. However, the authors claimed a gap that standard DL models require supervision by a large amount of labeled data, while self-supervised models often require an analytical model characterizing the underlying sensing process. Motivated by this, the work aims to propose a general self-supervised deep learning method, called Deep Empirical Neural Network (DENN), which combines the pre-computed empirical physical model and the trainable neural network. In this way, DENN bridges the claimed gap and offer better signal retrieval performance.

General Evaluation: The core contribution of the work is the proposal of DENN. At a high level, DENN can be viewed as a variant of the deep image prior (DIP), where the trainable network is used to generate the image and the empirical model is used to simulate the measurements of the generated image for computing the difference from the true measurements. Similar methods include [1-3]. While superior reconstruction performance of DENN over supervised models was demonstrated, the results are not surprising and have been shown in [1].

First, it is difficult to set a methodological difference between [1-3] and DENN. The use of either an analytical or empirical physical model would not change the overall formulation of the method. It is unclear why replacing an analytical physical model with an empirical model can be viewed as the methodological novelty. Second, the performance of DENN seems to heavily rely on the accuracy of the empirical model.
While current results demonstrate good performance of DENN, it is unclear how DENN would generalize to cases where the empirical model is less accurate (cor. = {0.9, 0.75, 0.5}). Furthermore, some baseline methods are missing, placing difficulties to interpret superiority of DENN.

The issue of methodological novelty is further negatively emphasized by the current narrative of the manuscript. The authors aimed to pitch DENN as a general DL method, but only one application to MMF is presented in the experiment, which is not enough to support the story. The title has the same problem. Given the critique on the novelty of DENN, it is suggested to lower down the tune of the paper.

In general, DENN seems to be very similar to the existing DIP-type methods [1-3]. The methodological novelty on the use of empirical physical model is debatable.

------------- Technical Comments-----------

1. What is the difference between the analytic and empirical models? It is not clear how to differentiate these two types of models. For example, linear scattering models can be viewed as empirical models as they cannot account for the entire light propagation process. Additionally, it is not clear why using an empirical model would pose challenges in algorithm design, because it plays the same role as the analytic model in DIP-type methods. In the current manuscript, these points are not addressed; hence, the motivation of DENN is not convincing.

2. As I did not see the difference of using an empirical model, DENN seems to be a reinvention of the methods proposed in [1-3]. The authors should carefully compare DENN with [1-3], especially [1].

3. I guess the empirical model needs to be re-calibrated for each different fiber. This seems largely hinders the application of DENN. This limitation needs to be addressed.

4. How would DENN function with inaccurate empirical models? My understanding is that the performance of DENN would decrease with lower accuracy. Experiments on various accuracy levels needs to be provided for understanding the generalizability of DENN. Additionally, the current experiments only consider the MMF of 10m length. It is also suggested to validate DENN on MMF with various length, see [4].

5. The current experiments missed some important baseline methods. For instance, plug-and-play priors [5] and regularization by denoising [6] are also proposed as effective methods for combining physical models and deep neural networks. Without a side-by-side comparison, it is unclear if DENN achieves the state-of-the-art performance.

6. Does the simulated MMF measurements contain extra noise, e.g. Gaussian noise? It desired to see whether DENN is robust to different noise scenarios.

References

[1] Phase imaging with an untrained neural network
[2] Deep phase decoder: self-calibrating phase microscopy with an untrained deep neural network
[3] Compressive sensing with un-trained neural networks: Gradient descent finds a smooth approximation
[4] Learning to see through multimode fibers
[5] Regularized Fourier ptychography using an online plug-and-play algorithm
[6] prDeep: Robust phase retrieval with a flexible deep network

(Remarks on code availability)

Reviewer #3

(Remarks to the Author)
The authors propose a concept of deep empirical neural network (DENN) that is a hybridization of a deep neural networks (DNN) and an empirical model. The empirical model captures the relationship between multiple inputs and their corresponding outputs for the scattering system, which can be described by empirical transmission matrix (TM) and calibrated by the optical experimental system. Unlike an analytical theory that can predict the full evolution of the system for any given input, this model resembles a partial experimental measurement of the system, providing insights for the update of DENN. Compared to the supervised learning, the DENN dramatically reduces the data amount and computational time by four and two orders of magnitudes, respectively, all while delivering over 48% improvement in fidelity, providing new light on the applications of deep learning in physics and information science. However, before endorsing publication I would like to point out the following comments that need to be addressed:

1. The empirical TM calibration and measurement process used in the experiment may have some inaccuracies, which can affect the accuracy of the final results. In some conditions, for example, as described in the first paragraph of the Discussion Section, the measurement of empirical TM for a strongly scattering medium cannot be perfect because of residual experimental noises. How does the author plan to minimize these impacts? How sensitive is the result to noise and outliers?

2. What optimization suggestions do the authors have for scalability issues that DENN approach may face when dealing with larger datasets or more complex problems, or when deploying across different environments and platforms?

3. The Pearson correlation coefficient (PCC)/structural similarity index measure (SSIM) under experiment shown in Fig. 3 is smaller than that under simulation. What does the difference originate from? The author should analyze the reasons in detail and propose improvement suggestions.

4. In the third paragraph of page 4, the authors believe that increasing the ratio γ can result in a better fidelity evaluated by PCC and SSIM. Why is there a significant difference between simulation SSIM and experiment SSIM in Fig. S3a of the supplementary materials? What are the main factors? What suggestions do the author have for improvement?

5. In the third paragraph of page 4, the authors found that SSIMloss is a suitable loss function in this case. What are the advantages of SSIM loss function compared to mean absolute error (MAE) loss functon(refer to Fig. S3b)?

(Remarks on code availability)
The supplementary code are reproducible for readers.

Version 1:

Reviewer comments:

Reviewer #1

(Remarks to the Author)
I would like to thank the authors for their detailed responses to the previous questions. The authors have provided comprehensive responses to the reviewers' comments, enhancing their manuscript by adding new experimental data, clarifying the presentation, and further elaborating on the innovation and superiority of the DENN method. Additionally, they addressed concerns about the method's robustness, scalability, and comparisons with other approaches through additional analyses and discussions, aiming to demonstrate the efficiency and applicability of DENN in optical phase retrieval tasks, especially for light propagation through scattering media. However, after carefully rereading the manuscript and all the reviewer comments and responses from the authors, I still find some unclear issues as follows.

1. The DENN proposed in this paper can deal with the problem of optical phase retrieval over a scattering medium. However, although the authors have explained the distinctions between DENN and DIP methods, DENN is still viewed as a variant of DIP for optical phase retrieval. Concretely, the authors emphasize that the DIP methods have been reported (for example, reference [33]) fail to deal with the problem of optical phase retrieval over a scattering medium, which can be attributed to the difference between diffraction optics and scattering optics. Further, the authors claim that comparing with the DIP methods with analytical theory which can only be applied in the scenario where optical scattering is absent, DENN can deal with the multiple scattering problems and be generalized to the homogeneous case.
However, like DIP methods have been reported, DENN can be regarded as physically driven deep neural network approach based on DIP, which combine with an untrained deep neural network and physical image formation model. Therefore, DIP methods have been reported and DENN differ only in the physical image formation model, such as the analytical model and the empirical model described in this paper. And the empirical model is described by an experimentally calibrated empirical transmission matrix, which bridges the input-output relationship.
In conclusion, the DENN proposed in this paper can be regarded as a physically driven DIP method for optical phase

retrieval, which can still align with previous DIP frameworks.

2. In the Supplementary Note 4, the comparison between the conventional supervised deep neural network and the empirical deep neural network still remains conceptually confused. Specifically, in the inference time component, the inference time for supervised learning is the sum of measured across the entire test set (0.48 seconds for a single speckle pattern and ~ 10 min for the entire test set), while for DENN, it is measured for a single speckle pattern (270 seconds for a single speckle pattern). This difference can cause conceptual confusion and lead to unfair comparisons. The authors should provide more details about the computational efficiency of the DENN algorithm, such as the time required to process a single sample.

3. Is it possible to optimize the calibrated empirical transmission matrix (TM) as a learnable parameter during the training of the Deep Empirical Neural Network (DENN)? Additionally, how does the TM influence the model's performance, and what challenges and considerations arise from its integration? How does the transmission matrix model deal with nonlinear effects in multiple scattering media? Are there more complex scenarios that haven't been considered?

4. In the comparison between the DENN and traditional methods, could the authors further detail the parameter analysis, especially explaining why DENN outperforms traditional methods in specific scenarios? Compare DENN with other existing optical phase retrieval techniques to demonstrate its relative advantages and potential limitations.

(Remarks on code availability)

Reviewer #2

(Remarks to the Author)
I thank the authors for carefully addressing my comments by providing clear explanations, revising the manuscript and title, and conducting extra experiments. The quality and clarity of the revised manuscript has been significantly improved, and most of my previous concerns have been sufficiently addressed. Before recommending the manuscript for publication, I have three minor comments to be discussed with the authors.

1.["However, the analytical models can only be applied in the scenario where optical scattering is absent."] To avoid potential misinterpretations, the authors are suggested to revise this sentence because analytic models for strong scattering have been investigated in the context of diffraction tomography, see [1,2].
2.[Regularization by Denoising] The RED method compared in the experiments is not the exact algorithm proposed in [3]. In fact, the so-called RED method is a combination of RED and DIP. The authors are suggested to rename the method to better reflect this difference.
3.To further strengthen the result, a comparison between DENN and the exact algorithm in [3] is recommended, because the recovery performance reported in [3] seems not to be consistent with what has been reported in the revised manuscript.

1. A Learning approach to optical tomography.
2. High-resolution 3D refractive index microscopy of multiple-scattering samples from intensity images.
3. prDeep: Robust Phase Retrieval with a Flexible Deep Network.

(Remarks on code availability)

Reviewer #3

(Remarks to the Author)
The authors made accurate responses to all my comments and suggestions. By adding new experimental results and descriptions, the rigor and completeness of this manuscript has been remarkably improved. Therefore, I suggest this manuscript to be accepted.

(Remarks on code availability)

Version 2:

Reviewer comments:

Reviewer #1

(Remarks to the Author)
I would like to thank the authors for their detailed responses to the previous questions. The explanations and extra experiments about the previous questions have been provided. Theoretical analysis and extensive experiments prove the technical correctness and effectiveness of DENN. However, as the authors acknowledge, the DENN proposed in this paper can be regarded as a physically driven DIP method for optical phase retrieval, which can still align with previous DIP frameworks for optical phase retrieval. Further, compared to analytical model, the authors have illustrated the advantages

and physical significance of empirical model.

(Remarks on code availability)

Reviewer #2

(Remarks to the Author)
I thank the authors for further addressing my previous concerns. I have no more comments on the manuscript.

(Remarks on code availability)

Open Access This Peer Review File is licensed under a Creative Commons Attribution 4.0 International License, which permits use, sharing, adaptation, distribution and reproduction in any medium or format, as long as you give appropriate credit to the original author(s) and the source, provide a link to the Creative Commons license, and indicate if changes were made.
In cases where reviewers are anonymous, credit should be given to 'Anonymous Referee' and the source.
The images or other third party material in this Peer Review File are included in the article’s Creative Commons license, unless indicated otherwise in a credit line to the material. If material is not included in the article’s Creative Commons license and your intended use is not permitted by statutory regulation or exceeds the permitted use, you will need to obtain permission directly from the copyright holder.
To view a copy of this license, visit https://creativecommons.org/licenses/by/4.0/

%%%%%%%%%%%%%%%%%%%%%%%%%%%%%
Response to the Referee 1
%%%%%%%%%%%%%%%%%%%%%%%%%%%%%

Blue color outlines the original text,

Red color indicates the corresponding modifications

GENERAL COMMENT 1:

The manuscript presents a method that combines Deep Image Prior (DIP) and the four-step phase-shifting method to address the inverse problem in a Multiple-Input Multiple-Output(MIMO) scattering system. The topic of work is interesting, and the authors provide a new perspective on addressing the inverse problem in MIMO scattering systems. However, I cannot recommend the manuscript to be published with current form. Following major comments should be addressed well.

OUR REPLY:

We really thank the Referee for pointing out that "The topic of work is interesting" and "the authors provide a new perspective on addressing the inverse problem in MIMO scattering systems". We would also like to thank the Referee for giving us an opportunity to further improve the quality of our manuscript.

All these very important comments of the Referee help us to substantially improve the quality of our manuscript. We address his/her valuable comments one by one in the following.

TECHNICAL COMMENT 1:

Clarification of Innovation:

The proposed method, while integrating two existing techniques, does not appear to fully explain its innovative aspects. The authors are advised to discuss in more detail the novelty of DENN compared to existing technologies, especially the theoretical or methodological innovations made in the process of combining DIP and the four-step phase-shifting method.

OUR REPLY:

We thank the Referee for this very important comment which helps us to clarify the novelty of our manuscript. We discuss the motivations of the DENN in the following three points:

1. The methodological motivation made in the integration of DIP and the four-step phase-shifting method is the embedding of the calibrated empirical transmission matrix into the topological structure of the neural network. Specifically, the empirical transmission matrix is embedded as an equivalent fully connected layer of the neural network, where the weights of the fully connected layer are the components of the matrix. It provides empirical guidance for updating the neural network, enabling optical phase retrieval over a strongly scattering medium in an untrained manner.
2. The DIP-type untrained neural networks reported so far for achieving optical phase retrieval using analytical theories cannot be used in the optical scattering systems considered in this manuscript. This is because these theories can only be adopted in the scenarios without

scattering, i.e. diffraction in homogenous media. In contrast, the DENN is quite general and it can be applied to various kinds of media, including scattering media and homogeneous media. Therefore, it is the DENN that enables the optical phase retrieval over a scattering medium in an untrained manner. Furthermore, we provide one more example for optical phase retrieval over an opaque ground glass, as shown in the Fig. R1. The retrieved PCC/SSIM of natural scene images using the DENN is as high as 0.94/0.78 and the retrieved accuracy of binary information is as high as 99.8%, showing good generality of the DENN for achieving optical phase retrieval in scattering optics.

[Image redacted]

Fig. R1 Experiment retrieval results of phase encoded natural scene images and uncorrelated binary information over an opaque ground glass (DG10-1500, Thorlabs). The ground truths, the measured output speckle patterns and the retrieved information of DENN are provided, respectively. The corresponding SSIM, PCC and bit accuracies are presented. The natural scene images are adapted from the ImageNet dataset [Proc. IEEE Conf. Comput. Vis. Pattern Recognit., 248-255 (2009)], where they are all 256-level gray scale images with a resolution of 64X64. The corresponding resolutions of speckle patterns are 384X384. The resolutions of the binary information are 32X 32 and their corresponding resolutions of speckle patterns are 256X256. Colorbars are also provided.

[Image redacted]

Fig. R2 Comparison of performances between the DENN and the speckle-correlation scattering matrix method over a 1km multimode fiber. The ground truths, the measured output speckle patterns of the MMF and the retrieved information of DENN and SSM are provided, respectively. The corresponding PCC/SSIM for the natural scene images and bit accuracy for the uncorrelated binary information are indicated, respectively. The natural scene images are adapted from the ImageNet dataset [Proc. IEEE Conf. Comput. Vis. Pattern Recognit., 248-255 (2009)], where they are 256-level grayscale images with a resolution of 64X64. Their corresponding resolutions of the speckle patterns are 384X384. While the resolutions of the binary information are 32X32 and

their corresponding resolutions of speckle patterns are 256 ×256. Colorbars are also provided.

3. We also perform quantitative comparison with the single-shot optical phase retrieval using the state-of-the-art speckle-correlation scattering matrix [Nat. Commun. 7, 13359 (2016)], which is also based on the transmission matrix. The comparison experiments are performed over a much longer multimode fiber (1km) than our previous results (10m). As shown in Fig. R2, the retrieved optical phase over a 1km multimode fiber by the scattering-correlated matrix method is not good. In contrast, the retrieved PCC/SSIM of natural scene images using the DENN is as high as 0.97/0.76 and the retrieved accuracy of binary information using the DENN is as high as 99.2% under the same experiment conditions, showing good superiority of the DENN for optical phase retrieval over a scattering medium.

According to these new experimental results, we try to convince the Referee that the unique embedding of the empirical model into the structure of the DIP-type neural network enables the realization of phase retrieval over a scattering medium. The results for the opaque ground glass (Fig. R1) and the 1km multimode fiber (Fig. R2) validate the generality of the DENN. While the results compared with the speckle-correlation scattering matrix (Fig. R2) show the superiority of the DENN.

In order to address this very important comment, we revise the sentences "However, there are many systems that lacks of analytical theory[44], which renders the physics-enhanced DNN based on the analytical theory inapplicable in these scenarios." to "However, the analytical models can only be applied in the scenario where optical scattering is absent[33,34]. There are many optical systems that lack of analytical theory[52], where wave scattering systems are typical examples. It renders the physics-enhanced DNN based on the analytical theory inapplicable in these scenarios. Therefore, leveraging the DIP concept for achieving optical phase retrieval over a scattering medium remains an alluring but challenging prospect."

"Essentially, the empirical TM for updating the DENN can be regarded as a fully connected (FC) layer with fixed weights right after the convnet, as indicated in Fig. 1c." is changed to "Essentially, the empirical TM for updating the DENN can be regarded as a fully connected (FC) layer with fixed weights right after the convnet, effectively describing the MIMO scattering process of the scattering medium. Therefore, it can be integrated into the topological structure of the DNN, as schematically indicated in Fig. 1c.".

The Figs. R1 and R2 are added to the Supplementary Information as a new Supplementary Notes 5 and 6, respectively, where the following discussions are also added in the Supplementary Information:

"We perform quantitative comparison between the single-shot optical phase retrieval using the state-of-the-art method of speckle-correlation scattering matrix (SSM)[4] and the DENN over a much longer MMF (1km, step index, diameter is 200 μm, NA = 0.22, YOFC). The SSM method is also based on the empirical TM. As shown in supplementary Figure 4, the retrieval results of the SSM method are not good over the 1km MMF by using a near infrared laser (λ = 850 nm, Toptica). In contrast, the retrieved PCC/SSIM of natural scene images using the DENN is as high as 0.97/0.76 and the retrieved accuracy of binary information using the DENN is as high as 99.2% under the

same experiment conditions, showing good superiority of the DENN for optical phase retrieval over a scattering medium.”

“To demonstrate the generality of DENN, we study the optical phase retrieval over the other scattering medium, i.e. an opaque ground glass, where the experiment results are shown in the supplementary Figure 5. The retrieved PCC/SSIM of natural scene images using the DENN is as high as 0.94/0.78 and the retrieved accuracy of binary information is as high as 99.8%, showing good generality of the DENN for achieving optical phase retrieval over different scattering media.”

TECHNICAL COMMENT 2:

Comparison of Data and Time:

The paper claims a reduction in the amount of training data and time, but DENN is essentially an unsupervised learning method. Comparing it with traditional supervised learning DNNs may require more consideration. The authors are encouraged to provide comparisons with traditional unsupervised or self-supervised learning methods to more accurately demonstrate the advantages of DENN.

OUR REPLY:

Yes, the Referee is right. The previous comparison is not rigorous. The fair comparison should be made by the same type of learning method. We revise the relevant statements to interpret the performances of different learning methods rather than direct comparison.

[Image redacted]

Fig. R3 Performance of optical phase retrieval over a 10m multimode fiber using the self-supervised learning method. The ground truths, the measured output speckle patterns of the MMF and the retrieved information of DENN are provided, respectively. The corresponding PCC/SSIM for the natural scene images is indicated. The natural scene images are adapted from the ImageNet dataset [Proc. IEEE Conf. Comput. Vis. Pattern Recognit., 248-255 (2009)], where they are 256-level grayscale images with a resolution of 64×64. Their corresponding resolutions of the speckle patterns are 384×384. Colorbars are also provided.

At the same time, we cannot find unsupervised learning method reported so far to achieve optical phase retrieval over a strongly scattering medium. In order to address this very important comment, we modify a physics-enhanced self-supervised learning method used in homogeneous media [Nat. Mach. Intell. 5, 895-907 (2023)] to fit the scattering scenarios considered in our manuscript, where the empirical model is embedded into the neural network. As shown in Fig. R3, the serf-supervised

learning method using 10000 data pairs cannot retrieve the input information of optical phase over a 10m multimode fiber with the training time of 78 hours. As shown in Fig. 3 (b) of our manuscript, the DENN succeed in retrieve the optical phase with high fidelity (PCC/SSIM ~ 0.97/0.84) under the same experiment conditions. Based on the new experimental results of optical phase retrievals over a ground glass using DENN (Fig. R1), comparison with speckle-correlation scattering matrix based on transmission matrix in a 1km long multimode fiber (Fig. R2) and self-supervised learning method (Fig. R3), it is shown that the proposed DENN does show advantages of achieving optical phase retrieval over a scattering medium.

In order to address this very important comment, we add the following sentence "We also study the integrating of the empirical model to the self-supervised learning model used in homogeneous media[25] and regulation by denoising[66,67] (Supplementary Notes 9 and 10), respectively, where the results are not as good as the DENN. Future efforts that combine the advantages of self-supervised learning model[25], regulation by denoising[66,67], plug-and-play priors[68] and DENN can be used to further improve the fidelity of optical phase retrieval over scattering media.".

The sentence "Both the substantial reductions of required data amount and computational time showcase the excellent performance of the proposed DENN." is changed to "The generalizations of DENN to the scenarios of 1km MMF (see Supplementary Note 5) and an opaque ground glass (see Supplementary Note 6) showcase the excellent performance of the proposed DENN for optical phase retrieval over a scattering medium."

The sentences "These experimental results validate that the required data amount of the DENN is four orders of magnitude less than the supervised case with even better fidelity. At the same time, the update process of the DENN takes about 270 s using the same computer hardware, which is two orders of magnitude less than the fastest supervised case." are deleted.

We also add the quantitative comparison with the modified self-supervised neural network as a new Supplementary Note 9, where the following discussions are also added in the Supplementary Information:

"A self-supervised learning scheme[5] used in homogeneous media is adapted to deal with the problem of optical phase retrieval over a scattering medium. A TM calibrated for a 10m MMF is used as the empirical model. 12500 unlabeled speckle patterns are used where they are divided into training set, verification set and test set in a ratio of 8:1:1. $SSIM_{loss}$ and AdamW are still used as loss functions and optimizers for the self-supervised network, respectively. After 40 epochs and 78 hours of training, the retrieved average PCC/SSIM of natural scene images of the test set is 0.779/0.307, as shown in supplementary Figure 8."

Two new references are added:
66. Metzler, C., Schniter, P., Veeraraghavan, A. & Baraniuk, R. prdeep: Robust phase retrieval with a flexible deep network. In *International Conference on Machine Learning*, 3501-3510 (PMLR, 2018).
68. Sun, Y. et al. Regularized fourier ptychography using an online plug-and-play algorithm. In

ICASSP 2019-2019 IEEE International Conference on Acoustics, Speech and Signal Processing (ICASSP), 7665-7669 (IEEE, 2019).

- -

TECHNICAL COMMENT 3:

Accuracy of Time Measurement:

When discussing performance improvements, comparing the iterative inference time of the network with training time may not be appropriate. The authors should clarify the distinction between training time and inference time and use accurate terminology in the discussion to avoid confusion.

OUR REPLY:

Yes, the Referee is right. Comparing the iterative inference time of the network with training time is not appropriate. We revise all the inappropriate statements, as shown in the following:

In order to address this comment, the sentence "Herein, we propose a concept of deep empirical neural network (DENN) that is a hybridization of a DNN and an empirical model. It enables seeing through an opaque scattering medium in an untrained manner. Compared to the supervised learning, the DENN dramatically reduces the data amount and computational time by four and two orders of magnitudes, respectively, all while delivering over 48% improvement in fidelity." in Abstract is changed to "Herein, we propose a concept of deep empirical neural network (DENN) that is a hybridization of a DNN and an empirical model, which enables seeing through an opaque scattering medium in an untrained manner. The DENN does not rely on labeled data, all while delivering as high as 48% improvement in fidelity compared with the supervised learning using 30000 data pairs for achieving the same goal of optical phase retrieval."

The sentences "General natural scene images that is too diverse to be precisely retrieved using data-driven DNN can be explicitly retrieved utilizing the DENN, achieving retrieved fidelity more than 48% better than the data-driven case. Compared to the data-driven DNN, the required data amount and the corresponding computational time for the neural network are reduced by four and two orders of magnitude, respectively." are changed to "General natural scene images that is too diverse to be precisely retrieved using data-driven DNN can be explicitly retrieved utilizing the DENN, where the highest retrieved fidelity is more than 48% better than the data-driven case (30000 data pairs). The training time and inference time of the data-driven DNN are about 26 hours and 0.48 second, respectively. While the DENN consumes 270 seconds in inference for a given speckle."

The sentence "Both the substantial reductions of required data amount and computational time showcase the excellent performance of the proposed DENN." is changed to "The generalizations of DENN to the scenarios of 1km MMF (see Supplementary Note 5) and an opaque ground glass (see Supplementary Note 6) showcase the excellent performance of the proposed DENN for optical phase retrieval over a strongly scattering medium."

The sentence "The required data amount and computational time are substantially reduced by four and two orders of magnitudes compared with the supervised DNN, respectively, even possessing more than 48% improvement of fidelity." is changed to "Considering the supervised learning using

30000 data pairs, the training time and inference time are about 26 hours and 0.48 second, respectively. While the DENN achieve the same goal of optical phase retrieval using the inference time of 270 seconds, all while delivering over 48% improvement in fidelity for a given natural scene image encoded information."

The "Time" shown in previous Supplementary Table 1 is divided into "Training time" and "Inference time", respectively, as shown in Table R1:

Comparison Items	Supervised deep neural network			Deep empirical neural network
Empirical model	N/A			**Empirical TM**
Data type	ImageNet			
Data amount	Train: 10000 pairs<br>Val: 1250 pairs<br>Test: 1250 pairs	Train: 20000 pairs<br>Val: 2500 pairs<br>Test: 2500 pairs	Train: 30000 pairs<br>Val: 3750 pairs<br>Test: 3750 pairs	**1 speckle pattern**
Training time	~ 08 hours 34 min	~ 17 hours 13 min	~ 25 hours 50 min	N/A
Inference time	~ 10 min	~ 21 min	~ 32 min	**~ 4 min 30s**
Averaged PCC/SSIM	0.787/0.456	0.825/0.506	0.840/0.524	**0.940/0.678**

Table R1 Comparison between the conventional supervised deep neural network and the empirical deep neural network

---

TECHNICAL COMMENT 4:

Adequacy of Comparative Experiments:

The paper requires more comparative experiments to demonstrate the superiority of the proposed method. The authors are advised to consider comparing with other existing technologies or methods, especially in terms of performance under different conditions and the application effects on different types of scattering media or inverse problems.

OUR REPLY:

We would like to thank the Referee for this very important comment and suggestion.

Following the suggestion of the Referee, we perform comparative experiments to demonstrate the superiority of the proposed method.

1. As can be seen from Fig. R2 presented above, the DENN shows substantial improvement of fidelity compared with the state-of-the-art speckle-correlation scattering matrix method [Nat. Commun. 7, 13359 (2016)] over a 1km long multimode fiber, validating the superiority of the DENN.

2. Comparing with the experimental results over a 10m long multimode fiber (Fig. 3b of our manuscript), the achieved fidelity of the 1km long multimode fiber is only slightly reduced, as shown in the left panel of Fig. R2. These results validate the robustness of the DENN under different conditions.

3. We perform a new experiment on a different type of scattering medium (an opaque ground glass) using the DENN. As can be seen from Fig. R1, similar fidelity as the multimode fiber scenarios is achieved, validating the generality of the DENN under different types of scattering media.

In order to address this very important comment, we add new experiments to demonstrate the superiority of the proposed method. These new experiments are added to the Supplementary Information as new Supplementary Notes 5 and 6, where the following discussions are also added in the Supplementary Information:

"We perform quantitative comparison between the single-shot optical phase retrieval using the state-of-the-art method of speckle-correlation scattering matrix (SSM)[4] and the DENN over a much longer MMF (1km, step index, diameter is 200 μm, NA = 0.22, YOFC). The SSM method is also based on the empirical TM. As shown in supplementary Figure 4, the retrieval results of the SSM method are not good over the 1km MMF by using a near infrared laser (λ = 850 nm, Toptica). In contrast, the retrieved PCC/SSIM of natural scene images using the DENN is as high as 0.97/0.76 and the retrieved accuracy of binary information using the DENN is as high as 99.2% under the same experiment conditions, showing good superiority of the DENN for optical phase retrieval over a scattering medium."

"To demonstrate the generality of DENN, we study the optical phase retrieval over the other scattering medium, i.e. an opaque ground glass, where the experiment results are shown in the supplementary Figure 5. The retrieved PCC/SSIM of natural scene images using the DENN is as high as 0.94/0.78 and the retrieved accuracy of binary information is as high as 99.8%, showing good generality of the DENN for achieving optical phase retrieval over different scattering media."

\- -

TECHNICAL COMMENT 5:
Comparison with Related Work:
It is suggested that the authors provide a more comprehensive discussion of related work in the literature review, especially those studies that are similar to or related to the DENN method.

OUR REPLY:
We would like to thank the Referee for this very important comment. We revise the discussion of DIP-type related work in more details.

In order to address this comment, we change the sentences to "Therefore, there is a growing pursuit of developing untrained DNN for acquiring the mapping function of unknown systems. Inspiring by the concept of deep image prior[32], physics-enhanced DNN has been proposed to overcome the challenge of big data by integrating physical prior into the neural network[33-39]. A typical physical-enhanced DNN for solving inverse problem relies on a forward mapping function[34], where an analytical model describing the physical evolution process is required[25, 28, 34]. The weights and biases of the physics-enhanced DNN can be updated using a loss function of analytical theory based physical consistence. It is quite efficient in dealing with the ill-posed problem of phase retrieval with solo intensity measurement[34, 35, 40-43], where the phase retardation can be precisely described

by a theory. However, there are many systems that lacks of analytical theory[44], which renders the physics-enhanced DNN based on the analytical theory inapplicable in these scenarios." to "Therefore, there is a growing pursuit of developing untrained DNN for acquiring the mapping function of unknown systems, where physics-enhanced DNN has been proposed to overcome the challenge of big data by integrating physical prior into the neural network[32-47]. Inspiring by the concept of deep image prior (DIP)[48], untrained neural network has been widely used in metrology[4], holography[36,42,43,46,47], imaging[29,39,40,44,45]. In particular, there are several variants of the DIP for optical phase retrieval in diffraction optics[33,34,39,49], where physics consistency is forced by minimizing the loss function between the estimated results and the actual measurement. Typical DIP-type DNNs for solving this inverse problem relies on a forward mapping function[33,34] to generate the estimated results, where an analytical model describing the physical evolution process is required[25,28,33]. The weights and biases of the physics-enhanced DNN can be updated using a loss function of analytical theory. It is quite efficient in dealing with the ill-posed problem of phase retrieval with solo intensity measurement[33,34,39,49-51], where the phase retardation can be precisely described by a theory. However, the analytical models can only be applied in the scenario where optical scattering is absent[33,34]. There are many optical systems that lack of analytical theory[52], where wave scattering systems are typical examples. It renders the physics-enhanced DNN based on the analytical theory inapplicable in these scenarios. Therefore, leveraging the DIP concept for achieving optical phase retrieval over a scattering medium remains an alluring but challenging prospect."

The following new references are added, where the citing sequence is updated throughout the manuscript:

36. Bai, C. et al. Dual-wavelength in-line digital holography with untrained deep neural networks. *Photonics Res.* 9, 2501-2510 (2021).

40. Chen, Q., Huang, D. & Chen, R. Fourier ptychographic microscopy with untrained deep neural network priors. *Opt. Express* 30, 39597-39612 (2022).

42. Galande, A. S., Thapa, V., Gurram, H. P. R. & John, R. Untrained deep network powered with explicit denoiser for phase recovery in inline holography. *Appl. Phys. Lett.* 122 (2023).

43. Liu, K., Wu, J., He, Z. & Cao, L. 4k-dmdnet: diffraction model-driven network for 4k computer-generated holography. *Opto-Electron. Adv.* 6, 220135-1 (2023).

44. He, Y. et al. Untrained neural network enhances the resolution of structured illumination microscopy under strong background and noise levels. *Adv. Photonics Nexus* 2, 046005 (2023).

45. Jin, Y. et al. Neural-field-assisted transport-of-intensity phase microscopy: partially coherent quantitative phase imaging under unknown defocus distance. *Photonics Res.* 12, 1494-1501 (2024).

46. Wang, Y. et al. Unsupervised deep learning enables 3d imaging for single-shot incoherent holography. *Laser Photonics Rev.* 2301091 (2024).

47. Huang, Z. & Cao, L. Quantitative phase imaging based on holography: trends and new perspectives. *Light. Sci. Appl.* 13, 145 (2024).

TECHNICAL COMMENT 6:

Strengthening of the Conclusion:

The conclusion section should provide a more comprehensive summary of the main contributions of DENN and discuss its potential for future research and practical applications.

OUR REPLY:

We thank the Referee for this comment. In order to address this comment, we change the sentences "We demonstrate a concept of DENN for seeing through an opaque medium without analytical theory, where the phase-encoded information scrambled by an MMF can be retrieved using a intensity-only measurement. Combining the precise empirical TM with the convnet provides an effective paradigm shift for unscrambling the multiple scattering of the MMF in an untrained manner. General natural scene images that is too diverse to be precisely retrieved using data-driven DNN can be explicitly retrieved utilizing the DENN, achieving retrieved fidelity more than 48% better than the data-driven case. Compared to the data-driven DNN, the required data amount and the corresponding computational time for the neural network are reduced by four and two orders of magnitude, respectively. It should be emphasized that the measurement of empirical TM for a strongly scattering medium cannot be perfect because of residual experimental noises[55]. If the precision of the calibrated TM can be further increased[56,57], the performance of the DENN can be further improved. By numerically generating TMs with Gaussian distributions that can mimic the physical properties of various strongly scattering media[58], the DENN can also retrieve general natural scene images and uncorrelated binary information with high accuracy (see Supplementary Note 5 for more details). These results indicate that the proposed DENN is general and can be applied for various scattering media whose empirical TM can be measured. It can enable the high fidelity phase retrieval with only a single-shot intensity measurement, providing a potential solution for the general but long standing challenging in optical communication, optical metrology, and holography. It is anticipated that the DENN can not only provide new insight for the black box problem of strongly scattering media, but also shed new light on much more broader disciplines of unknown systems relied on the data-driven DNN[59,60]." to "We demonstrate a concept of DENN for seeing through an opaque medium where the untrained neural network using analytical theory is not applicable. By integrating the empirical model that functions as an equivalent FC layer with a convnet, the phase-encoded information scrambled by MMFs with different lengths and the opaque ground glass can be retrieved in an untrained manner, providing an effective paradigm shift for unscrambling the multiple scattering. General natural scene images that is too diverse to be precisely retrieved using data-driven DNN can be explicitly retrieved utilizing the DENN, where the highest retrieved fidelity is more than 48% better than the data-driven case (30000 data pairs). The training time and inference time of the data-driven DNN are about 26 hours and 0.48 second, respectively. While the DENN consumes 270 seconds in inference for a given speckle. The DENN can also be generalized to the homogeneous case, while the analytical theory-based methods[33,34] cannot be applied in the scattering scenarios. It should be emphasized that the measurement of empirical TM for a strongly scattering medium cannot be perfect because of residual experimental noises[63]. Though the DENN is quite robust to noise, the performance of the DENN can be improved if the precision of the calibrated TM can be further increased[64,65]. We also study the integrating of the empirical model to the self-supervised learning model used in homogeneous media[25] and regulation by denoising[66,67] (Supplementary Notes 9 and 10), respectively, where the results are not as good as the DENN. Future efforts that combine the advantages of self-supervised learning model[25], regulation by denoising[66,67], plug-and-play priors[68] and DENN can be used to further improve the

fidelity of optical phase retrieval over scattering media. Dynamical learning can also be used to improve the robustness to vibration[69]. By numerically generating TMs with Gaussian distributions that can mimic the physical properties of various strongly scattering media[70], it is shown that the DENN can retrieve general natural scene images and uncorrelated binary information with high accuracy over different scattering media (see Supplementary Note 11 for more details). These results indicate that the proposed DENN is general and can be applied for various scattering media whose empirical TM can be measured. It can enable the high fidelity phase retrieval with only a single-shot intensity measurement, providing a potential solution for the general but long standing challenging in optical communication, optical metrology, and holography, where various transmission media suffer from unwanted scattering. It is anticipated that the DENN can not only provide new insight for the black box problem of strongly scattering media, but also shed new light on much more broader disciplines of unknown wave scattering systems relied on the data-driven DNN[71,72].".

Three new references are added:

66. Metzler, C., Schniter, P., Veeraraghavan, A. & Baraniuk, R. prdeep: Robust phase retrieval with a flexible deep network. In *International Conference on Machine Learning*, 3501-3510 (PMLR, 2018).

68. Sun, Y. et al. Regularized fourier ptychography using an online plug-and-play algorithm. In *ICASSP 2019-2019 IEEE International Conference on Acoustics, Speech and Signal Processing (ICASSP)*, 7665-7669 (IEEE, 2019).

69. Li, Z., Zhou, W., Zhou, Z. *et al.* Self-supervised dynamic learning for long-term high-fidelity image transmission through unstabilized diffusive media. *Nat Commun* **15**, 1498 (2024).

In summary, we really thank the Referee for these important comments which substantially help us to improve the quality of our manuscript.

In order to further convince the Referee, we perform two new experiments of optical phase retrieval over an opaque ground glass and 1km scale multimode fiber to show the generality of the deep empirical neural network. We also perform two new comparative experiments with the self-supervised neural network and speckle-correlation scattering matrix to show the superiority of the proposed concept. All these new results consolidate that the DENN enables the optical phase retrieval over a scattering medium, where the DIP-type neural networks reported so far cannot achieve.

It is anticipated that the revised manuscript is now can meet the criteria of Nature Communication and your consideration is sincerely appreciated.

%%%%%%%%%%%%%%%%%%%%%%%%%%%%%%%

Response to the Referee 2

%%%%%%%%%%%%%%%%%%%%%%%%%%%%%%%

Blue color outlines the original text

Red color indicates the corresponding modifications

- -

GENERAL COMMENT 1:

Problem, Motivation & Goal: This work considers the problem of retrieving signals from scattering measurements. Specifically, the considered signal is a 2D image(phase) and the measurements are the intensity of the light after transmitting a multi-mode fiber (MMF). Deep learning (DL) is a desired tool for solving the problem. However, the authors claimed a gap that standard DL models require supervision by a large amount of labeled data, while self-supervised models often require an analytical model characterizing the underlying sensing process. Motivated by this, the work aims to propose a general self-supervised deep learning method, called Deep Empirical Neural Network(DENN), which combines the pre-computed empirical physical model and the trainable neural network. In this way, DENN bridges the claimed gap and offer better signal retrieval performance.

OUR REPLY:

We really thank the Referee for recognizing that out that our work is "DENN bridges the claimed gap and offer better signal retrieval performance". We would like to ask the Referee for giving us an opportunity to improve the quality of our manuscript.

We are very sorry for our exaggerated statements in the manuscript. In our revised manuscript, we delete our exaggerated statements and revise our claim to be more realistic, which will be elaborated in the following.

To further convince the Referee, we perform two new experiments to support the proposed concept, where optical phase retrieval over a 1km length multimode fiber and an opaque ground glass are demonstrated. At the same time, we provide three new quantitative comparisons with baseline methods to prove the superiority and generality of our method. We also show two examples of perturbation effects on the estimated speckle pattern and calibrated empirical model to show the robustness of our method. All these new results consolidate that the DENN enables the optical phase retrieval over a scattering medium in an untrained manner, where deep image prior and its variants reported so far cannot achieve. Thank you again for these very valuable comments. Detail replies to general and technical comments are addressed one-by-one as follow:

- -

GENERAL COMMENT 2:

General Evaluation: The core contribution of the work is the proposal of DENN. At a high level, DENN can be viewed as a variant of the deep image prior (DIP), where the trainable network is used to generate the image and the empirical model is used to simulate the measurements of the generated image for computing the difference from the true measurements. Similar methods include [1-3]. While superior reconstruction performance of DENN over supervised models was

demonstrated, the results are not surprising and have been shown in [1].

OUR REPLY:

Yes, the Referee is right. The references [1-3] pointed out by the Referee and our manuscript can be all regarded as different variants of the DIP at a high level, with different scopes of application.

However, the methods mentioned in [1-3] fail to deal with the problem of optical phase retrieval over a scattering medium. Especially, these works are focus on diffraction optics while the problem considered in our manuscript belongs to scattering optics. It is the unique combination of empirical model and the DIP like process that can unscramble strongly scattering in an untrained manner, which is the main methodology novelty of the proposed concept.

The results of our manuscript haven't been shown in [1] pointed out by the Referee. The methodology of [1] relied on an analytical theory describing diffraction in uniform media. Because the theory can only be used in a homogeneous medium without scattering, it cannot treat the multiple scattering problems presented in our manuscript, such as a multimode fiber and an opaque ground glass (new added experiment results). While the empirical model we used can describe the multiple-input-multiple-output scattering process of scattering media, which can be effectively embedded in the neural network's topological structure as a fully connected layer. It should be pointed out that the empirical model can also be generalized to the homogeneous case shown in [1] while the method in [1] cannot be applied in the scenarios of this manuscript. Therefore, the proposed concept is different from the ones in [1] in the methodology of simulating the measurements of the intermediate output of the neural network, enabling the optical phase retrieval over a scattering medium. More importantly, we shown in the revised manuscript that the performance of the neural network is robust when the empirical model is added with moderate noise (see the following Rely to GENERAL COMMENT 3), consolidating the superiority and robustness of the proposed DENN.

In order to address this comment, we revise the sentences "However, there are many systems that lacks of analytical theory[44], which renders the physics-enhanced DNN based on the analytical theory inapplicable in these scenarios." to "However, the analytical models can only be applied in the scenario where optical scattering is absent[33,34]. There are many optical systems that lack of analytical theory[52], where wave scattering systems are typical examples. It renders the physics-enhanced DNN based on the analytical theory inapplicable in these scenarios. Therefore, leveraging the DIP concept for achieving optical phase retrieval over a scattering medium remains an alluring but challenging prospect." for strengthening the novelty of our manuscript.

The following sentence "The DENN can also be generalized to the homogeneous case, while the methods of diffraction optics[33,34] cannot be applied in the scattering scenarios." is added.

The sentence "Essentially, the empirical TM for updating the DENN can be regarded as a fully connected (FC) layer with fixed weights right after the convnet, as indicated in Fig. 1c." is changed to "Essentially, the empirical TM for updating the DENN can be regarded as a fully connected (FC) layer with fixed weights right after the convnet, effectively describing the MIMO scattering process of the scattering medium. Therefore, it can be integrated into the topological structure of the DNN,

as schematically indicated in Fig. 1c."

GENERAL COMMENT 3:

First, it is difficult to set a methodological difference between [1-3] and DENN. The use of either an analytical or empirical physical model would not change the overall formulation of the method. It is unclear why replacing an analytical physical model with an empirical model can be viewed as the methodological novelty. Second, the performance of DENN seems to heavily rely on the accuracy of the empirical model. While current results demonstrate good performance of DENN, it is unclear how DENN would generalize to cases where the empirical model is less accurate (cor. = {1, 0.9,0.75, 0.5}). Furthermore, some baseline methods are missing, placing difficulties to interpret superiority of DENN.

OUR REPLY:

We would like to thank the Referee for this very important comment, which help us to further distinguish our work with previous works. We address these comments in the following three points:

1. Replacing the analytical model with an empirical physical model is very important for enabling optical phase retrieval over a strongly scattering medium. As we mentioned in the Reply to GENERAL COMMENT 2, the analytical model-based methods can only be applied in the scenario of homogeneous environment. The precondition for using the analytical theory of diffraction is light propagation without scattering. It means that the method shown in [1] cannot be applied in the scenarios of scattering media. The unique combination of empirical model with the neural network enables the realization of untrained neural network for optical phase retrieval over different scattering media.

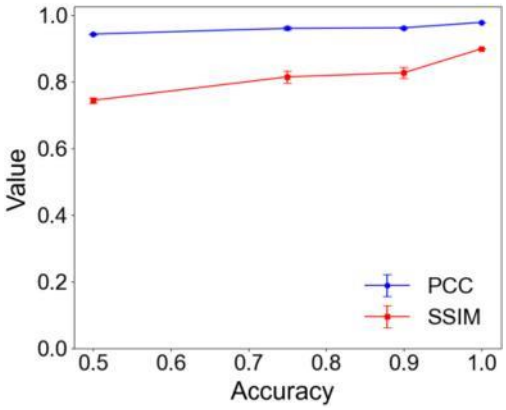

Fig. R4 The simulated dependences of retrieval fidelity on the accuracy of estimated speckle intensity. Here, the speckle accuracy is measured by PCC. The noise added in the speckle is Gaussian white noise. The resolution of the phase-encoded information is 64×64, The corresponding resolutions of speckle patters are 384×384. The PCC and SSIM are presented. The transmission matrix is calibrated in a 1m long multimode fiber.

2. Yes, the Referee is right. The performance of DENN is related to the accuracy of the empirical model. It should be emphasized out that the accuracy of transmission matrix based empirical model can be maintained higher than 0.9 in our experiment. We follow the Referee's suggestion to investigate the performance of DENN where the empirical model is less accurate by adding

noise to the estimated speckle patterns. As show in Fig. R4, the phase retrieval results of encoded natural scene image under four different speckle correlations (cor. = 1, 0.9, 0.75, 0.5) are provided. The PCC/SSIM is as high as 0.96/0.82 when the correlation is larger than 0.75. And the PCC/SSIM can be still higher than 0.94/0.74 when the correlation is equal to 0.5. These results inspired by the Referee indicates the DENN is quite robust to noise.

3. We further provide quantitative comparisons to three baseline methods, including regularization by denoising [International Conference on Machine Learning, 3501-3510. (PMLR, 2018)], self-supervising model [Nat. Mach. Intell. 5, 895-907 (2023)], and speckle-correlation scattering matrix based on transmission matrix [Nat. Commun. 7, 13359 (2016)] to interpret the superiority of DENN, where the results are shown in the following:

(1) Regularization by denoising: As can be seen from Fig. R5, by adding a regularization term, i.e.,

$\tilde{}\tilde{}_{2}(-\ {}^{2}{}_{*}\tilde{}())$ where λ is the regularization strength, to the loss function of Eq. (4) in our

manuscript, the performance of optical phase retrieval (λ = 0.05, 0.1, 0.2) is degraded compared with our case (λ = 0). By further optimizing the loss function with advanced regularization methods, the performance of DENN could be further improved.

[Image redacted]

Fig. R5 Simulation performance comparison between regularization by denoising (RED) and DENN. The ground truths, the estimated output speckle patterns of the MMF and the retrieved information of DENN and RED are provided, respectively. Here, three cases of RED (λ = 0.05, 0.1, 0.2) are provided. These images are adapted from the ImageNet dataset [Proc. IEEE Conf. Comput. Vis. Pattern Recognit., 248-255 (2009)], where they are all 256-level gray scale images with a resolution of 64×64. Their corresponding resolutions of the speckle patterns are 384×384. The corresponding SSIM and PCC are presented. Colorbars are also provided.

(2) Self-supervising model: we modify a physics-enhanced self-supervised learning method used in homogeneous media [Nat. Mach. Intell. 5, 895-907 (2023)] to fit the scattering scenarios considered in our manuscript, where the empirical model is integrated into the neural network. As shown in Fig. R6, the serf-supervised learning method using 10000 data pairs cannot retrieve the input information of optical phase over a multimode fiber with the training time of 78 hours. In contrast, the DENN succeed in retrieve the optical phase with high fidelity (PCC/SSIM ~ 0.97/0.84) over the same fiber, as shown in Fig. 3 (b) of our manuscript.

(3) Speckle-correlation scattering matrix: the quantitative comparison between the DENN and the

single-shot optical phase retrieval using the state-of-the-art speckle-correlation scattering matrix [Nat. Commun. 7, 13359 (2016)] is also performed. The speckle-correlation scattering matrix method is also based on the transmission matrix. The comparison experiments are performed over a much longer multimode fiber (1km) than our previous results (10m). As shown in Fig. R7, the fidelity of retrieved optical phase using the speckle-correlation scattering matrix method is not good. In contrast, the retrieved PCC/SSIM of natural scene images using the DENN is as high as 0.97/0.76 and the retrieved accuracy of binary information using the DENN is as high as 99.2% under the same experiment conditions.

These quantitative comparisons with three baseline methods further validate the superiority of the DENN for optical phase retrieval over a scattering medium.

[Image redacted]

Fig. R6 Performance of optical phase retrieval over a 10m multimode fiber using the self-supervised learning method. The ground truths, the measured output speckle patterns of the MMF and the retrieved information of DENN are provided, respectively. The corresponding PCC/SSIM for the natural scene images is indicated. Colorbars are also provided. The natural scene images are adapted from the ImageNet dataset [Proc. IEEE Conf. Comput. Vis. Pattern Recognit., 248-255 (2009)], where they are 256-level grayscale images with a resolution of 64X64. Their corresponding resolutions of the speckle patterns are 384X384.

[Image redacted]

Fig. R7 Comparison of performances between the DENN and the speckle-correlation scattering matrix method over a 1km multimode fiber. The ground truths, the measured output speckle patterns of the MMF and the retrieved information of DENN and SSM are provided, respectively. The corresponding PCC/SSIM for the natural scene images and bit accuracy for the uncorrelated binary information are indicated, respectively. The natural scene images are adapted from the ImageNet dataset [Proc. IEEE Conf. Comput. Vis. Pattern Recognit., 248-255 (2009)], where they are 256-level grayscale images with a resolution of 64X64. Their corresponding resolutions of the speckle patterns are 384X384. While the resolutions of the binary information are 32X32 and their corresponding resolutions of speckle patterns are 256X256. Colorbars are also provided.

In order to address this very important comments, we revise the sentences "However, there are many systems that lacks of analytical theory[44], which renders the physics-enhanced DNN based on the analytical theory inapplicable in these scenarios." to "However, the analytical models can only be applied in the scenario where optical scattering is absent[33,34]. There are many optical systems that lack of analytical theory[52], where wave scattering systems are typical examples. It renders the physics-enhanced DNN based on the analytical theory inapplicable in these scenarios. Therefore, leveraging the DIP concept for achieving optical phase retrieval over a scattering medium remains an alluring but challenging prospect."

The following sentences "Because the calibrated TM contained parasitic noise during measurement, the experimental retrieval results presented above take the noise into account. By artificially adding Gaussian white noise to the estimated speckle and empirical model, respectively, the performance of DENN is shown to be robust to noise (see Supplementary Notes 7 and 8 for the effects of noise on the performance of DENN)." are added.

The sentence "Future efforts that combine the advantages of self-supervised learning model[25], regulation by denoising[66,67], plug-and-play priors[68] and DENN can be used to further improve the fidelity of optical phase retrieval over scattering media." is added.

We also add the quantitative comparison with three baseline methods and the influence of accuracy of the estimated speckle pattern on the performance of the DENN as new Supplementary Notes 5, 7, 9, 10, where the following discussions are also added in the Supplementary Information:

"We perform quantitative comparison between the single-shot optical phase retrieval using the state-of-the-art method of speckle-correlation scattering matrix (SSM)[4] and the DENN over a much longer MMF (1km, step index, diameter is 200 μm, NA = 0.22, YOFC). The SSM method is also based on the empirical TM. As shown in supplementary Figure 4, the retrieval results of the SSM method are not good over the 1km MMF by using a near infrared laser (λ = 850 nm, Toptica). In contrast, the retrieved PCC/SSIM of natural scene images using the DENN is as high as 0.97/0.76 and the retrieved accuracy of binary information using the DENN is as high as 99.2% under the same experiment conditions, showing good superiority of the DENN for optical phase retrieval over a scattering medium."

"As shown in supplementary Figure 6, four scenarios of speckle accuracy (cor. = 0.5, 0.75, 0.9, 1) are studied by adding different amounts of Gaussian white noise to the estimated speckle pattern. It can be seen that the achieved PCC/SSIM is as high as 0.96/0.82 when cor. ≥ 0.75. Even when cor. = 0.5, the achieved PCC/SSIM is still higher than 0.94/0.74."

"A self-supervised learning scheme[5] used in homogeneous media is adapted to deal with the problem of optical phase retrieval over a scattering medium. A TM calibrated for a 10m MMF is used as the empirical model. 12500 unlabeled speckle patterns are used where they are divided into training set, verification set and test set in a ratio of 8:1:1. $SSIM_{loss}$ and AdamW are still used as loss functions and optimizers for the self-supervised network, respectively. After 40 epochs and 78 hours

of training, the retrieved average PCC/SSIM of natural scene images of the test set is 0.779/0.307, as shown in supplementary Figure 8."

"We also quantitatively compare performances between the regularization by denoising (RED)[6] and the DENN in simulation. First, we generate the training dataset by adding Gaussian white Noise (std = 30 dB) to 10,000 images from ImageNet[3]. We get the denoiser by utilizing DnCNN[7] to fit the training dataset. The $SSIM_{loss}$ and AdamW are still used as the loss functions and optimizer to minimize the following expressions:

$$_{*}(,) = arg\ min_{\tilde{\varphi},m} \| \, | \cdot^{1}() \cdot |_{2} - \| + _{2}\tilde{}^{2}\tilde{(} - {}^{2}{}_{*}(\tilde{}))$$

where F_m^1 is our neural network mapping the speckle intensity I to the phase $\tilde{}$, λ is the RED regularization strength, and $F_{m^{*2}}$ is the pre-trained denoising model. The corresponding comparison results are shown in supplementary Figure 9. The performances of optical phase retrieval (λ = 0.05, 0.1, 0.2) are degraded compared with our case (λ = 0)."

Two new references are added:

66. Metzler, C., Schniter, P., Veeraraghavan, A. & Baraniuk, R. prdeep: Robust phase retrieval with a flexible deep network. In *International Conference on Machine Learning*, 3501-3510 (PMLR, 2018).

68. Sun, Y. et al. Regularized fourier ptychography using an online plug-and-play algorithm. In *ICASSP 2019-2019 IEEE International Conference on Acoustics, Speech and Signal Processing (ICASSP)*, 7665-7669 (IEEE, 2019).

GENERAL COMMENT 4:

The issue of methodological novelty is further negatively emphasized by the current narrative of the manuscript. The authors aimed to pitch DENN as a general DL method, but only one application to MMF is presented in the experiment, which is not enough to support the story. The title has the same problem. Given the critique on the novelty of DENN, it is suggested to lower down the tune of the paper.

OUR REPLY:

We thank the Referee for this very important comment. We are very sorry for our exaggerated statement in our previous manuscript. The focus of this manuscript is the proposed DENN enables optical phase retrieval over a strongly scattering medium. According to the suggestions of the Referee, we lower down the tune of our manuscript. In order to show the generality of the DENN, we also perform a new experiment of optical phase retrieval in a different scattering medium.

The experiment results over an opaque ground glass are shown in Figs. R8. The typical PCC/SSIM for the natural scene image is up to 0.94/0.78 while the typical bit accuracy for the random binary information is up to 99.8%, which are as good as the cases of multimode fibers. These new experimental results showcase the generality and robustness of the proposed D

[Image redacted]

Fig. R8 Experiment retrieval results of phase encoded natural scene images and uncorrelated binary information over an opaque ground glass (DG10-1500, Thorlabs). The ground truths, the measured output speckle patterns and the retrieved information of DENN are provided, respectively. The corresponding SSIM, PCC and bit accuracies are presented. The natural scene images are adapted from the ImageNet dataset [Proc. IEEE Conf. Comput. Vis. Pattern Recognit., 248-255 (2009)], where they are all 256-level gray scale images with a resolution of 64X64. The corresponding resolutions of speckle patterns are 384X384. The resolutions of the binary information are 32X 32 and their corresponding resolutions of speckle patterns are 256X256. Colorbars are also provided.

In order to address this comment, the title of our manuscript is changed from "Deep empirical neural network" to "Deep empirical neural network for optical phase retrieval over a scattering medium" to focus on our main motivation.

The sentences "Inspiring by the concept of deep image prior[32], physics-enhanced DNN has been proposed to overcome the challenge of big data by integrating physical prior into the neural network[33-39]. A typical physical-enhanced DNN for solving inverse problem relies on a forward mapping function[34], where an analytical model describing the physical evolution process is required[25,28,34]. The weights and biases of the physics-enhanced DNN can be updated using a loss function of analytical theory based physical consistence. It is quite efficient in dealing with the ill-posed problem of phase retrieval with solo intensity measurement34, 35, 40-43, where the phase retardation can be precisely described by a theory. However, there are many systems that lacks of analytical theory[44], which renders the physics-enhanced DNN based on the analytical theory inapplicable in these scenarios." are changed to "Inspiring by the concept of deep image prior (DIP)[48], untrained neural network has been widely used in metrology[4], holography[36,42,43,46,47], imaging[29,39,40,44,45]. In particular, there are several variants of the DIP for optical phase retrieval in diffraction optics[33,34,39,49], where physics consistency is forced by minimizing the loss function between the estimated results and the actual measurement. Typical DIP-type DNNs for solving this inverse problem relies on a forward mapping function[33,34] to generate the estimated results, where an analytical model describing the physical evolution process is required[25,28,33]. The weights and biases of the physics-enhanced DNN can be updated using a loss function of analytical theory. It is quite efficient in dealing with the ill-posed problem of phase retrieval with solo intensity measurement[33,34,39,49-51], where the phase retardation can be precisely described by a theory.

However, the analytical models can only be applied in the scenario where optical scattering is absent[33,34]. There are many optical systems that lack of analytical theory[52], where wave scattering systems are typical examples. It renders the physics-enhanced DNN based on the analytical theory inapplicable in these scenarios. Therefore, leveraging the DIP concept for achieving optical phase retrieval over a scattering medium remains an alluring but challenging prospect."

The sentences "We demonstrate a concept of DENN for seeing through an opaque medium without analytical theory, where the phase-encoded information scrambled by an MMF can be retrieved using a intensity-only measurement. Combining the precise empirical TM with the convnet provides an effective paradigm shift for unscrambling the multiple scattering of the MMF in an untrained manner." are changed to "We demonstrate a concept of DENN for seeing through an opaque medium where the untrained neural network using analytical theory is not applicable. By integrating the empirical model that functions as an equivalent FC layer with a convnet, the phase-encoded information scrambled by MMFs with different lengths and the opaque ground glass can be retrieved in an untrained manner, providing an effective paradigm shift for unscrambling the multiple scattering.".

The sentence "Both the substantial reductions of required data amount and computational time showcase the excellent performance of the proposed DENN." is changed to "The generalizations of DENN to the scenarios of 1km MMF (see Supplementary Note 5) and an opaque ground glass (see Supplementary Note 6) showcase the excellent performance of the proposed DENN for optical phase retrieval over a scattering medium."

The new experimental results for an opaque ground glass are added as the new Supplementary Note 6, where the following discussions are also added in the Supplementary Information:

"To demonstrate the generality of DENN, we study the optical phase retrieval over the other scattering medium, i.e. an opaque ground glass, where the experiment results are shown in the supplementary Figure 5. The retrieved PCC/SSIM of natural scene images using the DENN is as high as 0.94/0.78 and the retrieved accuracy of binary information is as high as 99.8%, showing good generality of the DENN for achieving optical phase retrieval over different scattering media."

\- -

GENERAL COMMENT 5:

In general, DENN seems to be very similar to the existing DIP-type methods [1-3]. The methodological novelty on the use of empirical physical model is debatable.

OUR REPLY:

We thank the Referee for this very important comment. It is our unclear presentation that leads to this misunderstanding. The proposed concept enables the optical phase retrieval over a strongly scattering medium with high fidelity, which cannot be achieved using the existing DIP-type methods. We will address the following important technical comments one-by-one, which substantially help us to improve the quality of our manuscript.

In order to address this comment, the following sentence "The DENN can also be generalized to the homogeneous case, while the methods of diffraction optics[33,34] cannot be applied in the scattering scenarios." is added.

– –

TECHNICAL COMMENT 1:

What is the difference between the analytic and empirical models? It is not clear how to differentiate these two types of models. For example, linear scattering models can be viewed as empirical models as they cannot account for the entire light propagation process. Additionally, it is not clear why using an empirical model would pose challenges in algorithm design, because it plays the same role as the analytic model in DIP-type methods. In the current manuscript, these points are not addressed; hence, the motivation of DENN is not convincing.

OUR REPLY:

We thank the Referee for this very important comment which help us to clarify the novelty of our manuscript.

1. The difference between the analytic and empirical models lies in the generality of two methods. The reported analytical model can provide the link between the output intensity distribution and a given input phase before diffraction. However, there is strict requirement that the medium between the input and output planes is homogeneous, where no multiple scattering occurs during the diffraction. It means that the analytical model is not applicable in the scattering optics. There are many optical scattering systems that lack of analytical theory for describing the propagation of light [Nat. Phys. 18, 994-1007 (2022)]. In our case, the empirical model relies on the calibration of transmission matrix, which can account for the nonlocal effect of multiple-input-multiple-output resulted from the multiple scattering and possess clear physical meaning.
2. We are sorry for our unclear presentation which leads to the misunderstanding. All the DIP-type methods sharing similar mind of algorithm design. We didn't mean that using an empirical model would pose challenges in algorithm design. In our manuscript, we try to show that using an analytical model for untrained neural network poses challenge in optical phase retrieval over a scattering medium, where this scattering scenario is quite general in optical imaging, communication and detection. By integrating the empirical model into the neural network, the phase retrieval can be achieved from a seemly chaotic speckle pattern, providing an effective solution to this challenge in scattering optics.

In order to address this comment, we revise the following sentence "Because the combination of a neural network with the empirical TM is general and without any subsidiary condition, the DENN can be generalized for diverse inverse problems of information retrieval in black box systems without analytical theory." to "Because the empirical TM can be calibrated for different scattering media, the combination of a neural network with the empirical TM is applicative for various media. Therefore, the DENN can be generalized for diverse inverse problems of information retrieval in black box systems without analytical theory.".

The sentence "It can enable the high fidelity phase retrieval with only a single-shot intensity

measurement, providing a potential solution for the general but long standing challenging in optical communication, optical metrology, and holography." is changed to "It can enable the high fidelity phase retrieval with only a single-shot intensity measurement, providing a potential solution for the general but long standing challenging in optical communication, optical metrology, and holography, where various transmissive channels suffer from unwanted scattering."

– –

TECHNICAL COMMENT 2:

As I did not see the difference of using an empirical model, DENN seems to be a reinvention of the methods proposed in [1-3]. The authors should carefully compare DENN with [1-3], especially [1].

REPLY:

We thank the Referee for this important comment. As we mentioned above, the method shown in [1] cannot deal with the scattering problem. Because the empirical transmission matrix method is general and it can be applied in both homogeneous and inhomogeneous media. It means that the DENN can be used in the scenario demonstrated in [1] while the method shown in [1] cannot be used to the scenario of scattering medium.

In order to address this comment, we add a sentence "The DENN can also be generalized to the homogeneous case, while the methods of diffraction optics[33,34] cannot be applied in the scattering scenarios."

– –

TECHNICAL COMMENT 3:

I guess the empirical model needs to be re-calibrated for each different fiber. This seems largely hinders the application of DENN. This limitation needs to be addressed.

OUR REPLY:

Yes, the Referee is right. The empirical model needs to be re-calibrated for each different fiber. In our experiment, the acquisition time of the empirical model is about 1 second. The acquisition speed can be further increased by using high speed modulator and camara.

In order to address this comment, we add the following sentence "The empirical model should be re-calibrated for different fibers. A higher speed light modulator and a faster camera can be used to further improve the precision of measured empirical TM and reduce the acquisition time, simultaneously."

– –

TECHNICAL COMMENT 4:

How would DENN function with inaccurate empirical models? My understanding is that the performance of DENN would decrease with lower accuracy. Experiments on various accuracy levels needs to be provided for understanding the generalizability of DENN. Additionally, the current experiments only consider the MMF of 10m length. It is also suggested to validate DENN on MMF with various length, see [4].

OUR REPLY:

Yes, the Referee is right. The performance of DENN would decrease with lower accuracy. Because the calibrated empirical model naturally contains parasitic experimental noise, the demonstrated good performance of DENN is achieved in realistic environment with noise. Following the instruction of the Referee, we consider the scenarios with different accuracy (cor. = 1, 0.9, 0.75,0.5) by adding Gaussian white noise to the estimated speckle patterns. As can be seen from Fig. R4 in the Reply to GENERAL COMMENT 3, the PCC/SSIM of the phase retrieval using DENN can still higher than 0.96/0.82 when cor. ≥ 0.75, showing good robustness to noise.

To further convince the Referee, we perform optical phase retrieval experiments over a 1km long multimode fiber similar to the case of [4]. As can be seen from the results in Fig. R9, high fidelity phase retrievals for both the natural scene image and the uncorrelated binary information can be achieved, where much more complicated images are considered compared to [4].

[Image redacted]

Fig. R9 Experimental results of optical phase retrieval over a 1km multimode fiber using DENN. The ground truths, the measured output speckle patterns of the MMF and the retrieved information of DENN and SSM are provided, respectively. The corresponding PCC/SSIM and bit accuracy are indicated. The natural scene images are adapted from the ImageNet dataset [Proc. IEEE Conf. Comput. Vis. Pattern Recognit., 248-255 (2009)], where they are 256-level grayscale images with a resolution of 64X64. The corresponding resolutions of speckle patterns are 384X384. The resolution of the binary information is 32X32. While the resolutions of the speckle patterns are 256X256. Colorbars are also provided.

In order to address this very important comments, the following sentences "Because the calibrated TM contained parasitic noise during measurement, the experimental retrieval results presented above take the noise into account. By artificially adding Gaussian white noise to the estimated speckle and empirical model, respectively, the performance of DENN is shown to be robust to noise (see Supplementary Notes 7 and 8 for the effects of noise on the performance of DENN)." are added.

We also add the new experiment results of 1km multimode fiber as new Supplementary Note 5, where the corresponding discussion is also added:

"We perform quantitative comparison between the single-shot optical phase retrieval using the state-

of-the-art method of speckle-correlation scattering matrix (SSM)[4] and the DENN over a much longer MMF (1km, step index, diameter is 200 µm, NA = 0.22, YOFC). The SSM method is also based on the empirical TM. As shown in supplementary Figure 4, the retrieval results of the SSM method are not good over the 1km MMF by using a near infrared laser (λ = 850 nm, Toptica). In contrast, the retrieved PCC/SSIM of natural scene images using the DENN is as high as 0.97/0.76 and the retrieved accuracy of binary information using the DENN is as high as 99.2% under the same experiment conditions, showing good superiority of the DENN for optical phase retrieval over a scattering medium."

- -

TECHNICAL COMMENT 5:

The current experiments missed some important baseline methods. For instance, plug-and-play priors[5] and regularization by denoising [6]are also proposed as effective methods for combining physical models and deep neural networks. Without a side-by-side comparison, it is unclear if DENN achieves the state-of-the-art performance.

OUR REPLY:

We would like to thank the Referee for this very important comment, where quantitative comparisons with the state-of-the-art methods would be more straightforward evident for the superiority of the DENN.

The plug-and-play priors and regularization by denoising are indeed compatible methods to the proposed DENN rather than competitive methods. They can be used to facilitate the update of the untrained neural network by modulating the loss function of physics consistency. In order to convince the Referee, we provide quantitative comparison to the regularization by denoising [Int. Conf. Machine Learning, 3501-3510 (PMLR,2018)] to show the superiority of DENN. As can be seen from Fig. R5 mentioned in the Reply to GENERAL COMMENT 3, adding a regularization term to the loss function of Eq. (4) in our manuscript whose regularization term can be controlled by λ, the performances of optical phase retrieval (λ = 0.05, 0.1, 0.2) are degraded compared to the case without regularization (λ = 0). It should be also noted that we do not find any reference of integrating the empirical model into these baseline methods yet. By combing the advantages of plug-and-play priors [5] and regularization by denoising [6] with the DENN, the performance of optical phase retrieval could be further improved, which is out of the scope of our manuscript.

In order to address this very important comment, we add the quantitative comparison between the DENN and the regularization by denoising (Fig. R5 and its corresponding discussion) as a new Supplementary Note 10, where the following discussion is also added:

"We also quantitatively compare performances between the regularization by denoising (RED)[6] and the DENN in simulation. First, we generate the training dataset by adding Gaussian white Noise (std = 30 dB) to 10,000 images from ImageNet[3]. We get the denoiser by utilizing DnCNN[7] to fit the training dataset. The $SSIM_{loss}$ and AdamW are still used as the loss functions and optimizer to minimize the following expressions:

$$ {}_{*}(,) = arg\ min_{\tilde{\varphi}, m} \| \, | \cdot {}^{1}() \cdot |_2 - \| + {}_2 \tilde{(} - {}^{2}{}_{*} \tilde{(})) ^{2} $$

where F_m^1 is our neural network mapping the speckle intensity I to the phase $\tilde{}$, λ is the RED regularization strength, and $F_{m^{*2}}$ is the pre-trained denoising model. The corresponding comparison results are shown in supplementary Figure 9. The performance of optical phase retrieval (λ = 0.05, 0.1, 0.2) are degraded compared with our case (λ = 0).”

We also add the following sentences “Future efforts that combine the advantages of self-supervised learning model[25], regulation by denoising[66,67], plug-and-play priors[68] and DENN can be used to further improve the fidelity of optical phase retrieval over scattering media.”

Two new references are added:

66. Metzler, C., Schniter, P., Veeraraghavan, A. & Baraniuk, R. prdeep: Robust phase retrieval with a flexible deep network. In *International Conference on Machine Learning*, 3501-3510 (PMLR, 2018).

68. Sun, Y. et al. Regularized fourier ptychography using an online plug-and-play algorithm. In *ICASSP 2019-2019 IEEE International Conference on Acoustics, Speech and Signal Processing (ICASSP)*, 7665-7669 (IEEE, 2019).

TECHNICAL COMMENT 6:

Does the simulated MMF measurements contain extra noise, e. g. Gaussian noise? It desired to see whether DENN is robust to different noise scenarios.

OUR REPLY:

We thank the Referee for this inspired comment. The simulated MMF measurements do not contain extra noise. In order to address this inspired comment, we add different amounts of extra Gaussian white noise to the measured transmission matrix to study the robustness of DENN, where the signal-to-noise-ratio (SNR) of the transmission matrix is modified artificially. As can be seen from the results in Fig. R10, the phase retrieval fidelity of the DENN is quite robust to different amounts of noise whose SNR is varied from 5-100.

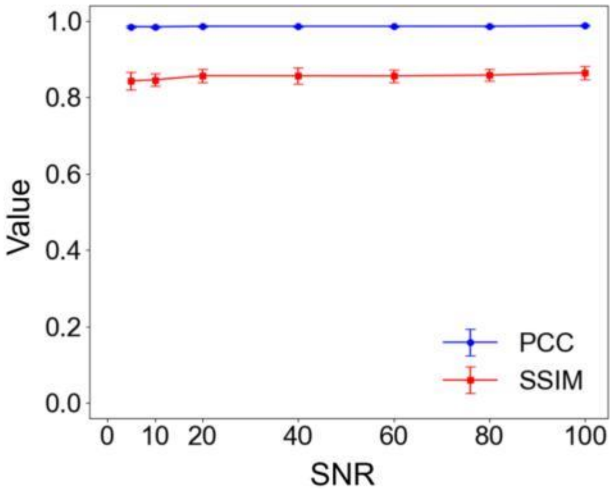

Fig. R10 Effects of signal-to-noise ratio (SNR) on the performance of DENN in simulation using

measurement transmission matrix. The resolution of the phase-encoded information is 64×64 and the corresponding resolutions of speckle patterns are 384×384, The PCC and SSIM are presented. 6 realizations are used for each SNR, where the error bars are also provided.

In order to address this comment, we add the following sentences "Because the calibrated TM contained parasitic noise during measurement, the experimental retrieval results presented above take the noise into account. By artificially adding Gaussian white noise to the estimated speckle and empirical model, respectively, the performance of DENN is shown to be robust to noise (see Supplementary Notes 7 and 8 for the effects of noise on the performance of DENN)."

We also add the influence of noise on the performance of DENN as a new Supplementary Note 8, where the following discussion is also added:

"We quantitatively evaluate the performance of DENN when the empirical TM is perturbed by different amounts of noise in simulation. Different amounts of Gaussian white noise are added to the empirical TM, where the corresponding SNR varies between 5 and 100 (SNR = 5, 10, 20, 40, 60, 80, 100). From the results shown in supplementary Figure 7, it can be seen that the phase retrieval fidelity of DENN is robust to different levels of noise."

In summary, we really thank the Referee for these very important comments which substantially help us to improve the quality of our manuscript. We are very sorry for the exaggerated statements in our previous manuscript, where such claims are revised to be more realistic. The title is also modified to focus on the functionality of DENN.

The new experimental results of a 1km long multimode fiber and an opaque ground glass are presented to consolidate our claim. The quantitative comparisons between DENN and three baseline methods are used to demonstrate the superiority of DENN. Two examples of perturbing the estimated speckle patterns and calibrated empirical model are used to show the robustness of our method. All these new results consolidate that the DENN enables the optical phase retrieval over a scattering medium, where the reported DIP-type methods cannot achieve.

It is anticipated that the revised manuscript is now can meet the criteria of Nature Communication and your reconsideration is sincerely appreciated.

%%%%%%%%%%%%%%%%%%%%%%%%%%%%%%%%%

Response to the Referee 3

%%%%%%%%%%%%%%%%%%%%%%%%%%%%%%%%%

Blue color outlines the original text

Red color indicates the corresponding modifications

- -

GENERAL COMMENT:

The authors propose a concept of deep empirical neural network (DENN) that is a hybridization of a deep neural networks (DNN) and an empirical model. The empirical model captures the relationship between multiple inputs and their corresponding outputs for the scattering system, which can be described by empirical transmission matrix (TM) and calibrated by the optical experimental system. Unlike an analytical theory that can predict the full evolution of the system for any given input, this model resembles a partial experimental measurement of the system, providing insights for the update of DENN. Compared to the supervised learning, the DENN dramatically reduces the data amount and computational time by four and two orders of magnitudes, respectively, all while delivering over 48% improvement in fidelity, providing new light on the applications of deep learning in physics and information science. However, before endorsing publication I would like to point out the following comments that need to be addressed:

OUR REPLY:

We would like to thank the Referee for pointing out that our results "providing new light on the applications of deep learning in physics and information science" and giving us an opportunity to further improve the quality of our manuscript.

We would also like to thank the Referee for giving us very important comments which help us to substantially shape our manuscript. We address his/her major comments one by one in the following.

- -

COMMENT 1:

The empirical TM calibration and measurement process used in the experiment may have some inaccuracies, which can affect the accuracy of the final results. In some conditions, for example, as described in the first paragraph of the Discussion Section, the measurement of empirical TM for a strongly scattering medium cannot be perfect because of residual experimental noises. How does the author plan to minimize these impacts? How sensitive is the result to noise and outliers?

OUR REPLY:

We thank the Referee for this very important and inspiring comment.

Because our experimental results essentially contain parasitic experimental noise, the DENN is robust to the realistic noisy system. In order to further demonstrate the robustness of the DENN, we conduct a new experiment where the optical phase retrieval is achieved over a 1km long multimode

fiber, which is more sensitive to the environment than the 10m case shown in our previous manuscript. As can be seen from the results in Fig. R11, the achieved fidelity is as good as the 10m one [Fig. 3 (b) in the main text].

Because the perturbations from environment (vibration and change of temperature) is the main noise in experiment, the most straightforward way to improve the precision of TM measurement is becoming faster in TM calibration. It means that the precision of empirical TM measurement can be improved by using a faster light modulator and a faster camara. Now the measured time for the TM mentioned in our manuscript is about 1 second, which can also be further reduced by optimizing the dimensions of TM.

[Image redacted]

Fig. R11 Experimental results of optical phase retrieval over a 1km multimode fiber using DENN. The ground truths, the measured output speckle patterns of the MMF and the retrieved information of DENN and SSM are provided, respectively. The corresponding PCC/SSIM and bit accuracy are indicated. Colorbars are also provided. The natural scene images are adapted from the ImageNet dataset [Proc. IEEE Conf. Comput. Vis. Pattern Recognit., 248-255 (2009)], where they are 256-level grayscale images with a resolution of 64X64. The corresponding resolutions of speckle patterns are 384X384. The resolution of the binary information is 32X32. While the resolutions of the speckle patterns are 256X256.

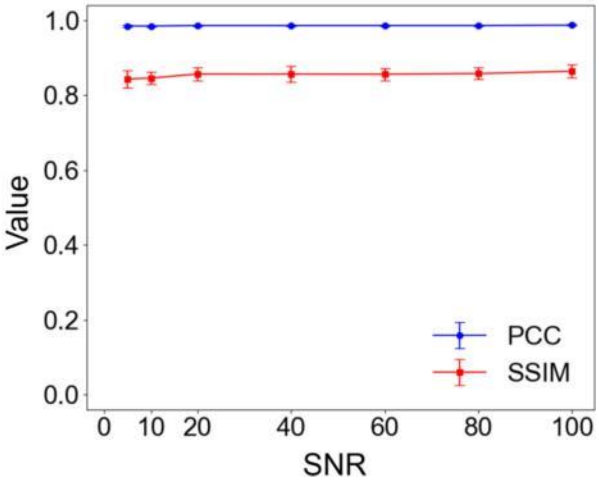

Fig. R12 Effects of signal-to-noise ratio (SNR) on the performance of DENN in simulation using measurement transmission matrix. The resolution of the phase-encoded information is 64X64 and the corresponding resolutions of speckle patterns are 384X384. The PCC and SSIM are presented. 6 realizations are used for each SNR, where the error bars are also provided.

We also investigate the influence of noise on the performance of optical phase retrieval. Different amounts of Gaussian white noise are artificially added to the measured transmission matrix to study the robustness of DENN over noise. As can be seen from the results in Fig. R12, the phase retrieval fidelity of the DENN is quite robust to different amounts of noise whose signal-to-noise-ratio (SNR) is varied from 5-100. These results consolidate that the DENN is robust to noise.

In order to address this comment, we add the following sentences "Because the calibrated TM contained parasitic noise during measurement, the experimental retrieval results presented above take the noise into account. By artificially adding Gaussian white noise to the estimated speckle and empirical model, respectively, the performance of DENN is shown to be robust to noise (see Supplementary Notes 7 and 8 for the effects of noise on the performance of DENN). " and "A higher speed light modulator and a faster camera can be used to further improve the precision of measured empirical TM and reduce the acquisition time, simultaneously."

We also add the new experiment result of the 1km multimode fiber and the influence of additional noise added in the empirical transmission matrix on the performance of the DENN as new Supplementary Notes 5 and 8, where the following discussions are also added in the Supplementary Information:

"We perform quantitative comparison between the single-shot optical phase retrieval using the state-of-the-art method of speckle-correlation scattering matrix (SSM)[4] and the DENN over a much longer MMF (1km, step index, diameter is 200 μm, NA = 0.22, YOFC). The SSM method is also based on the empirical TM. As shown in supplementary Figure 4, the retrieval results of the SSM method are not good over the 1km MMF by using a near infrared laser (λ = 850 nm, Toptica). In contrast, the retrieved PCC/SSIM of natural scene images using the DENN is as high as 0.97/0.76 and the retrieved accuracy of binary information using the DENN is as high as 99.2% under the same experiment conditions, showing good superiority of the DENN for optical phase retrieval over a scattering medium."

"We quantitatively evaluate the performance of DENN when the empirical TM is perturbed by different amounts of noise in simulation. Different amounts of Gaussian white noise are added to the empirical TM, where the corresponding SNR varies between 5 and 100 (SNR = 5, 10, 20, 40, 60, 80, 100). From the results shown in supplementary Figure 7, it can be seen that the phase retrieval fidelity of DENN is robust to different levels of noise."

COMMENT 2:
What optimization suggestions do the authors have for scalability issues that DENN approach may face when dealing with larger databasets or more complex problems, or when deploying across different environments and platforms?

OUR REPLY:
We thank the Referee for this very important comment. Considering the problems with larger

datasets and more complex problems, the loss function of physical consistence can be modified to improve the performance of the DENN. For example, the advantages of the plug-and-play priors [Proc. IEEE Int. Conf. Acoust., Speech Signal Process., 7665-7669 (2019)] and regularization by denoising [Int. Conf. Machine Learning, 3501-3510 (PMLR,2018)] can be combined to improve the performance of DENN. Modifying the topological structure of the convnet is also an effective mean.

[Image redacted]

Fig. R13 Experiment retrieval results of phase encoded natural scene images and uncorrelated binary information over an opaque ground glass (DG10-1500, Thorlabs). The ground truths, the measured output speckle patterns and the retrieved information of DENN are provided, respectively. The corresponding SSIM, PCC and bit accuracies are presented. Colorbars are also provided. The natural scene images are adapted from the ImageNet dataset [Proc. IEEE Conf. Comput. Vis. Pattern Recognit., 248-255 (2009)], where they are all 256-level gray scale images with a resolution of 64X64. The corresponding resolutions of speckle patterns are 384X384. The resolutions of the binary information are 32X32 and their corresponding resolutions of speckle patterns are 256X 256.

In the revised manuscript, we also perform a new experiment for optical phase retrieval over an opaque ground glass to show the ability of DENN when deploying across different environments, where the results are shown in Fig. R13. The achieved PCC/SSIM is as good as the ones of multimode fibers. These results further validate the generalization and robustness of the proposed neural network.

In order to address this very important comment, we add the following sentences "Future efforts that combine the advantages of self-supervised learning model[25], regulation by denoising[66,67], plug-and-play priors[68] and DENN can be used to further improve the fidelity of optical phase retrieval over scattering media." in the main text.

We also add the new experiment of optical phase retrieval over an opaque ground glass to support the proposed concept, where the new experiment is added to the Supplementary Information as the new Supplementary Note 6, where the following discussion is also added in the Supplementary Information:

"To demonstrate the generality of DENN, we study the optical phase retrieval over the other

scattering medium, i.e. an opaque ground glass, where the experiment results are shown in the supplementary Figure 5. The retrieved PCC/SSIM of natural scene images using the DENN is as high as 0.94/0.78 and the retrieved accuracy of binary information is as high as 99.8%, showing good generality of the DENN for achieving optical phase retrieval over different scattering media." Two new references are added:

66. Metzler, C., Schniter, P., Veeraraghavan, A. & Baraniuk, R. prdeep: Robust phase retrieval with a flexible deep network. In *International Conference on Machine Learning*, 3501-3510 (PMLR, 2018).

68. Sun, Y. et al. Regularized fourier ptychography using an online plug-and-play algorithm. In *ICASSP 2019-2019 IEEE International Conference on Acoustics, Speech and Signal Processing (ICASSP)*, 7665-7669 (IEEE, 2019).

- -

COMMENT 3:

The Pearson correlation coefficient (PCC)/structural similarity index measure (SSIM) under experiment shown in Fig. 3 is smaller than that under simulation. What does the difference originate from? The author should analyze the reasons in detail and propose improvement suggestions.

OUR REPLY:

We thank the Referee for this very important comment. The reason why the PCC/SSIM achieved in experiment is smaller than that of simulation is because the speckle intensity I used in simulation is based on calculation, as indicated by Eq. (3) in the main text of our manuscript. In this case, the phase-encoded wavefront, TM and the estimated speckle are all perfect in principle. In experiment, the phase-encoded wavefront and calibration of TM are accomplished by a digital mirror device, where there is parasitic noise for the phase modulation of wavefront. At the same time, there is additional noise of capturing the speckle in experiment. These factors result in discrepancy of phase retrieval results between experiment and simulation. Improving the phase modulation precision of the digital mirror device and using a higher speed camera might reduce this difference.

In order to address this very important comment, we change the sentence "The slight reduction of bit accuracy for the experiment case is because of the imperfect empirical TM calibration." to "The slight reductions of fidelity and bit accuracy for the experiment cases compared with the simulation ones shown in Figs. 3 and 4 is because the generated phase wavefront and captured speckle are perfect in simulation. Improving the phase modulation precision of the DMD could reduce this discrepancy." to interpret the origin of difference and propose improvement suggestion.

We also add the following sentences "A higher speed light modulator and a faster camera can be used to further improve the precision of measured empirical TM and reduce the acquisition time, simultaneously."

- -

COMMENT 4:

In the third paragraph of page 4, the authors believe that increasing the ratio γ can result in a better fidelity evaluated by PCC and SSIM. Why is there a significant difference between simulation SSIM

and experiment SSIM in Fig. S3 a of the supplementary materials? What are the main factors? What suggestions do the author have for improvement?

OUR REPLY:

We would like to thank the Referee for this very comment and suggestion. Here, -y indicates the ratio between the resolutions of the speckle pattern and the phase-encoded information. Increasing the ratio -y can improve the precision of the calibrated TM and provide more information to the neural network simultaneously. The reason why there is a significant difference between the simulation SSIM and experiment SSIM especially for the case of the smallest ratio -y is similar to the one shown in the Reply to TECHNICAL COMMENT 3. The comparison results shown in Fig. 3 of our manuscript can be regarded as a specified case (-y = 36) in Fig. S3 (a) of the supplementary material. The phase-encoded wavefront, TM and speckle intensity for evaluating the loss function in simulation are without noise. While the generated phase-encoded wavefront, calibrated TM and speckle intensity for evaluating the loss function in experiment are imperfect. There is parasitic error in generating the targeted phase-encoded wavefront using the digital mirror device. The calibrated TM and the experimental recoded speckle are perturbed by the environment noise, which result in degraded results.

In order to address this very important comment, the sentence "As can be seen from this figure, increasing -y can dramatically enhance the performance of the DENN." is changed to "The phase-encoded wavefront, TM and speckle intensity for evaluating the loss function in simulation are perfect in principle. While there is parasitic noise in generated phase-encoded wavefront, calibrated TM and captured speckle, which result in the discrepancy between simulation and experiment. As can be seen from this figure, increasing -y can dramatically enhance the performance of the DENN, where the retrieval fidelity can be improved by selecting a suitable and available ratio -y." is added in the Supplementary Note 3.

We also add the following sentence "A higher speed light modulator and a faster camera can be used to further improve the precision of measured empirical TM and reduce the acquisition time, simultaneously."

COMMENT 5:

In the third paragraph of page 4, the authors found that SSIMloss is a suitable loss function in this case. What are the advantages of SSIM loss function compared to mean absolute error (MAE) loss function(refer to Fig. S3b)?

OUR REPLY:

We thank the Referee for this comment. Compared to the case of using MAE as the loss function, the advantage of using the SSIM_loss lies in its ability to not only consider pixel-level differences but also focus on the nonlocal structure, brightness, and contrast. Therefore, the SSIM value of the case using SSIM_loss is slightly higher than the MAE_loss case in the phase retrieval task of natural scene images, the SSIM_loss case can more effectively preserve the image's details and structural

characteristics, resulting in better information retrieval.

By optimizing SSIM_loss, the DENN can maintain the performance of optical phase retrieval while enhancing sensitivity to local features, avoiding the distortion issues that arise from solely optimizing pixel-level differences.

In order to address this comment, we change the sentence "It can be found that the case of using $SSIM_{loss}$ loss function possesses the best performance." to "It can be found that the case of using $SSIM_{loss}$ loss function possesses the best performance, because the $SSIM_{loss}$ can not only consider pixel-level differences but also focus on the nonlocal properties of the information." in Supplementary Notes 3.

COMMENT 6:
The supplementary codes are reproducible for readers.

OUR REPLY:
We thank the Referee for this important recognition.

In summary, we really thank the Referee for these important and inspiring comments which substantially help us to shape our manuscript.

We provide two new experimental results of a 1km long multimode fiber and an opaque ground glass in the revised manuscript to consolidate our claim. The DENN is also shown to be quite robustness to noise. All these new results consolidate that the DENN's superiority, generality, and robustness in dealing with the optical phase retrieval over a scattering medium.

It is anticipated that the revised manuscript is now can meet the criteria of Nature Communication and your consideration is sincerely appreciated.

Response letters

We would like to thank three Referees for their insightful and constructive comments on our manuscript, which have substantially helped us to improve the quality of our manuscript. We would also like to thank the Editor for giving us another opportunity to revise our manuscript.

Before addressing Referees' specific comments below, we would like to clarify the key motivation of our work. The central idea explored in this study is the combination of empirical model with neural network. Introducing the empirical model enables both the forward and backward propagations of the neural network under the guidance of realistic system response in an untrained manner. In particular, the differentiable empirical model with a matrix form naturally allows the back propagation of the neural network, which is crucial for achieving the untrained neural network for optical phase retrieval over a strongly scattering medium.

We have carefully considered all the comments raised from the Referees and addressed all the issues in the response letter. Four new experiments are performed to further strength the proposed concept. It is hoped that the revised version is much improved from the original one and your consideration is sincerely appreciated.

Please see our one-by-one responses below:

%%%%%%%%%%%%%%%%%%%%%%%%%%%%%%%%%%%%

Response to the Referee 1

%%%%%%%%%%%%%%%%%%%%%%%%%%%%%%%%%%%%

Blue color outlines the original text,

Red color indicates the corresponding modifications

GENERAL COMMENT:

I would like to thank the authors for their detailed responses to the previous questions. The authors have provided comprehensive responses to the reviewers' comments, enhancing their manuscript by adding new experimental data, clarifying the presentation, and further elaborating on the innovation and superiority of the DENN method. Additionally, they addressed concerns about the method's robustness, scalability, and comparisons with other approaches through additional analyses and discussions, aiming to demonstrate the efficiency and applicability of DENN in optical phase retrieval tasks, especially for light propagation through scattering media. However, after carefully rereading the manuscript and all the reviewer comments and responses from the authors, I still find some unclear issues as follows.

OUR REPLY:

We really thank the Referee for the very important comments in two rounds of review, which help us to substantially improve the quality of our manuscript. We also would like to thank the Referee for acknowledging "The authors have provided comprehensive responses to the reviewers' comments, enhancing their manuscript by adding new experimental data, clarifying the presentation, and further elaborating on the innovation and superiority of the DENN method."

All the valuable comments from the Referee do help us to improve the quality of our manuscript. We address all the comments one by one in the following.

TECHNICAL COMMENT 1:

The Deep Empirical Neural Network (DENN) proposed in this paper can deal with the problem of optical phase retrieval over a scattering medium. However, although the authors have explained the distinctions between DENN and DIP methods, DENN is still viewed as a variant of DIP for optical phase retrieval. Concretely, the authors emphasize that the DIP methods have been reported (for example, reference [33]) fail to deal with the problem of optical phase retrieval over a scattering medium, which can be attributed to the difference between diffraction optics and scattering optics. Further, the authors claim that comparing with the DIP methods with analytical theory which can only be applied in the scenario where optical scattering is absent, DENN can deal with the multiple scattering problems and be generalized to the homogeneous case. However, like DIP methods have been reported, DENN can be regarded as physically driven deep neural network approach based on DIP, which combine with an untrained deep neural network and physical image formation model. Therefore, DIP methods have been reported and DENN differ only in the physical image formation model, such as the analytical model and the empirical model described in this paper. And the empirical model is described by an experimentally calibrated empirical transmission matrix, which bridges the input-output relationship.

In conclusion, the DENN proposed in this paper can be regarded as a physically driven DIP method for optical phase retrieval, which can still align with previous DIP frameworks.

OUR REPLY:
Yes, the Referee is right. We really thank the Referee for this very important comment. Indeed, the proposed DENN can be regarded as a physically driven DIP method for optical phase retrieval with a different physical guiding method.

We will try to convince the Referee that introducing the empirical model using realistic system response could enable ① the back propagation and update of the neural network under the guidance of realistic physical response in an untrained manner, ② the consideration of optical backward scattering in optical phase retrieval, ③ increase the accuracy of physically driven effect of the neural network and ④ learnable parameters of the transmission matrix, as elaborated in the following four points:

1. Enabling the back propagation of the neural network under the guidance of realistic response in an untrained manner:
Back propagation is one of the most important features in deep neural networks. Previous works rely on differentiable analytical models to achieve untrained neural networks. In the optical phase retrieval of either diffraction optics and scattering optics, updating the neural network under the guiding of realistic response in an untrained manner is fundamentally limited by the difference between the experimental measured intensity and the output phase of the neural network. To achieve this goal, a differentiable empirical model is necessary.

Inspired by the comment of the Referee, we find that the most important difference of the DENN compared with previous DIP related works lies in the combination of the matrix-type empirical model and neural network, where the empirical model is mathematically differentiable for the back propagation of neural network. This empirical model naturally provides a mathematical link between the output phase of the neural network and the detected speckle intensity, enabling both forward and backward propagation of the neural network in an untrained manner. Specially, back propagation of the neural network started from the evaluation of loss function regarding to speckle intensity is enabled by the empirical model, fascinating the optical phase retrieval over a scattering medium in an untrained manner. Without the empirical model, guiding the neural network using realistic response of the system remains challenging. This could be an interesting point distinct from previous DIP related works, which is not guided by the real response of the system.

2. A different physically driven method that enables the consideration of optical backward scattering:
There are several works related to DIP considering optical phase retrieval in diffraction optics [Light. Sci. Appl. 9, 77 (2020); Optica 7, 559-562 (2020); Optica, 2015, 2, 517-522; Optica, 2019, 6, 12111219], where the diffraction process is modelled by either the analytical formula of Huygens – Fresnel principle or the beam propagation method [J. Opt. Soc. Am., 1981, 71, 803-810]. The loss function of physical consistence can be achieved using the numerically calculated diffraction pattern, resembling an elegant analytical physics guidance for realizing optical phase retrieval. In these

works, the optical forward scattering dominates and backward scattering can be neglected. However, as pointed out in these references, these methods can only deal with the optical systems with negligible backward scattering. In contrast, the empirical model used in our manuscript is fully adaptive to optical systems even with strong backward scattering. This argument is further supported by the experimental results using a ground glass and bended multimode fibers with different lengths (Figs. 3-5, Supplementary Fig. 4, Supplementary Fig. 5 and of the manuscript). The demonstrated performance is better than the supervised neural network and the state-of-the-art speckle-correlation scattering matrix method. Therefore, a new application scope of the DIP type methods is demonstrated.

3. The empirical model provides a better physical guiding accuracy than the analytical model: There are inevitable discrepancies between theoretical and realistic responses of the system. It means that the diffraction pattern numerically calculated for evaluating the physical consistency of loss function would deviate from the real response of the system. At the same time, the permittivity and permeability of the optical system cannot be perfect homogeneous. If the degree of inhomogeneous is increased, the physically driven accuracy based on the analytical model would degrade and the update of neural network might not be convergent. Furthermore, inaccurately estimating the length of optical path can also influence the accuracy of image calculated by the analytical model.

In contrast, the response of the system is described in an end-to-end manner using the empirical model, providing realistic and robust physical guidance for the optical phase retrieval. The embedding of empirical model to the neural network not only enables the optical phase retrieval over strong scattering media in an untrained manner, but also automatically integrates the real response of the system to achieve the physical consistency.

4. Learnable parameters of the transmission matrix proposed by the Referee.
As suggested by the Technical comment 3 of the Referee, the elements of the empirical model can become learnable, which could enable the update of physical model and improve the fidelity of optical phase retrieval guided by empirical physics consistence. We really thank the Referee for suggesting the concept of learnable transmission matrix and the Technical comment 3 will be addressed in the following Reply to Comment.

In order to address this very important comment, we add the following sentences "However, generating the estimated results using an empirical model relied on the real response of system, where the model should be differentiable for the back propagation of the neural network, resembles a promising while open attempt to update the neural network in an untrained manner." in the Introduction and "The embedding of empirical model to the neural network not only enables the optical phase retrieval over strong scattering media in an untrained manner, but also automatically integrates the real response of the system to facilitate the physical consistency under back propagation of the neural network." in the Discussion to the strengthen the novelty of this manuscript.

We also change the sentence from "However, the analytical models can only be applied in the

scenario where optical scattering is absent[33,34]. There are many optical systems that lack of analytical theory[52], where wave scattering systems are typical examples." to "Although beam propagation method[52] can account for weak scattering scenarios with negligible backward scattering[53,54], the analytical models can only be applied in the scenario where strongly optical scattering is absent[33,34]. Furthermore, there are many optical systems without analytical theory[55], where wave scattering systems with non-negligible backward scattering are typical examples."

The sentence "Elements in the TM can become learnable parameters of the DENN, which might further improve the achieved fidelity." is also added in the Discussion section.

The following references are added:

[52] Roey, J. V., van der Donk, J. & Lagasse, P. E. Beam-propagation method: analysis and assessment. J. Opt. Soc. Am. 71, 803-810 (1981).

[53] Kamilov, U. S. et al. Learning approach to optical tomography. Optica 2, 517 − 522 (2015).

[54] Chowdhury, S. et al. High-resolution 3D refractive index microscopy of multiple-scattering samples from intensity images. Optica 6, 1211-1219 (2019).

\- -

TECHNICAL COMMENT 2:

In the Supplementary Note 4, the comparison between the conventional supervised deep neural network and the empirical deep neural network still remains conceptually confused. Specifically, in the inference time component, the inference time for supervised learning is the sum of measured across the entire test set (0.48 seconds for a single speckle pattern and ~ 10 min for the entire test set), while for DENN, it is measured for a single speckle pattern (270 seconds for a single speckle pattern). This difference can cause conceptual confusion and lead to unfair comparisons. The authors should provide more details about the computational efficiency of the DENN algorithm, such as the time required to process a single sample.

OUR REPLY:

We would like to thank the Referee for this very important comment. Yes, the Referee is right. The comparison remains confused in the Supplementary Note 4. Although we did explicitly point out the training and inference time for the supervised learning and DENN in the main text of our last revision, respectively, as shown in the following:

"Considering the supervised learning using 30000 data pairs, the training time and inference time are about 26 hours and 0.48 second, respectively. While the DENN achieve the same goal of optical phase retrieval using the inference time of 270 seconds, all while delivering over 48% improvement in fidelity for a given natural scene image encoded information."

"General natural scene images that is too diverse to be precisely retrieved using data-driven DNN can be explicitly retrieved utilizing the DENN, where the highest retrieved fidelity is more than 48% better than the data-driven case (30000 data pairs). The training time and inference time of the data-driven DNN are about 26 hours and 0.48 second, respectively. While the DENN consumes 270 seconds in inference for a given speckle.", we did make a mistake in revising the Supplementary Table 1. As suggested by the Referee, in order to explicitly point out the time required to process a single sample, we revise the Supplementary Table 1 as follow:

Comparison Items	Supervised deep neural network			Deep empirical neural network
Empirical model	N/A			**Empirical TM**
Data type	ImageNet			
Data amount	Train: 10000 pairs<br>Val: 1250 pairs<br>Test: 1250 pairs	Train: 20000 pairs<br>Val: 2500 pairs<br>Test: 2500 pairs	Train: 30000 pairs<br>Val: 3750 pairs<br>Test: 3750 pairs	**1 speckle pattern**
Computational time for 1 speckle pattern	Train: ~ 08 hours 34 min<br>Inference: ~ 0.48 s	Train: ~ 17 hours 13 min<br>Inference: ~ 0.48 s	Train: ~ 25 hours 50 min<br>Inference: ~ 0.48 s	**Inference: ~ 4 min 30 s**
Averaged PCC/SSIM	0.787/0.456	0.825/0.506	0.840/0.524	**0.940/0.678**

Table R1 Comparison between the conventional supervised deep neural network and the empirical deep neural network

---

TECHNICAL COMMENT 3:

Is it possible to optimize the calibrated empirical transmission matrix (TM) as a learnable parameter during the training of the Deep Empirical Neural Network (DENN)? Additionally, how does the TM influence the model's performance, and what challenges and considerations arise from its integration? How does the transmission matrix model deal with nonlinear effects in multiple scattering media? Are there more complex scenarios that haven't been considered?

OUR REPLY:

We really thank the Referee for this VERY inspiring suggestion and comment. Yes, the elements in the calibrated empirical transmission matrix can become learnable parameters during the updating of the DENN. We choose the elements in 10 rows of the TM as learnable parameters to test the effectiveness of this idea. The choosing criterion is based on the mismatch between the estimated and captured speckles. As can be seen from Fig. R1, the structural similarity index measure (SSIM) can be improved from 0.724 to 0.733. The SSIM might be further improved by choosing more suitable elements in the TM as learnable parameters, which will be addressed in our future work. We would like to thank the Referee again for this VERY inspiring and interesting suggestion.

[Image redacted]

Fig. R1 The experimental retrieval results of a phase-encoded natural scene image through a 10m MMF (diameter is 105 μm, NA = 0.22, YOFC). The measured output speckle of the MMF, the estimated speckle using the empirical model, the optimized speckle when partial elements of the empirical model become learnable, and the retrieved information of DENN are provided, respectively. The corresponding SSIM and PCC are presented. Colorbars are also provided. This

image is adapted from the ImageNet dataset, where it is a 256-level gray scale image with a resolution of 64×64. The resolutions of all speckle patterns are 384×384.

Yes, the Referee is right. The performance of DENN is related to the accuracy of the empirical model. We have numerically investigated the performance of DENN when the TM is assumed to be less accurate by artificially reducing the correlation between the estimated and captured speckles. The phase retrieval results of an encoded natural scene image under different accuracy (speckle correlation = 1, 0.9, 0.75, 0.5) are shown in Fig. R2. The PCC/SSIM is as high as 0.96/0.82 when the correlation is larger than 0.75. It should be noted that the correlation for empirical physics guidance is higher than 0.9 in experiment.

We didn't consider nonlinear effect yet in our experiment because the laser we used is a CW laser and the media we use are not material with high optical nonlinearity. As can be seen from our experimental results, the works presented in this manuscript still belong to the region of linear optics. However, this comment is quite inspiring for us, which might inspire new applications. Whether this method can be generalized to the nonlinear case [Nat. Phys. 19, 1709-1718 (2023), Adv. Photonics, 5, 046010-046010 (2023)] is a very interesting prospect. We will address this very interesting idea in our future work.

Is it possible to used less information as the input of DENN to achieve optical phase retrieval with the same level of fidelity is a challenge as well as another scenario we didn't consider in the integration of empirical model and neural network. We will also try to address this scenario in our future work.

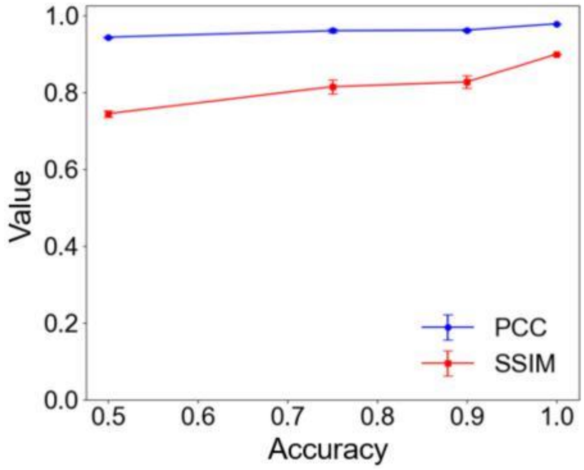

Fig. R2 The simulated dependences of retrieval fidelity on the accuracy of estimated speckle intensity. Here, the speckle accuracy is measured by PCC. The noise added in the speckle is Gaussian white noise. The resolution of the phase-encoded information is 64×64, The corresponding resolutions of speckle patters are 384×384. The PCC and SSIM are presented. The transmission matrix is calibrated in a 1m long multimode fiber.

In order to address this very important comment, we add three sentences "Elements in the TM can become learnable parameters of the DENN, which might further improve the achieved fidelity.", "Using a more lightweight neural network and reducing the size of speckle pattern can be used to

reduce the inference time of DENN." and "The nonlinear effects of the multiple scattering media are not considered yet[74,75] in this work, which could inspire new applications." in the Discussion section.

Fig. R2 is presented by the Supplementary Fig. 6.

The following references are added:

[74] Moon, J., Cho, Y.-C., Kang, S., Jang, M. & Choi, W. Measuring the scattering tensor of a disordered nonlinear medium. Nat. Phys. 19, 1709-1718 (2023).

[75] Ni F, Liu H, Zheng Y, et al. Nonlinear harmonic wave manipulation in nonlinear scattering medium via scattering-matrix method. Adv. Photonics, 5, 046010-046010 (2023).

TECHNICAL COMMENT 4:

In the comparison between the DENN and traditional methods, could the authors further detail the parameter analysis, especially explaining why DENN outperforms traditional methods in specific scenarios? Compare DENN with other existing optical phase retrieval techniques to demonstrate its relative advantages and potential limitations.

OUR REPLY:

Yes, the Referee is right. We need to further detail the parameter analysis when the performance of DENN is compared with the traditional method. In order to address this important comment, we further perform quantitative comparison with the state-of-the-art speckle-correlation scattering matrix (SSM) [Nat. Commun. 7, 13359 (2016)] under different parameters. As mentioned in this reference, a larger ratio (γ) between output and input channel numbers is crucial for increasing the fidelity of optical phase retrieval. Therefore, additional comparison experiments are performed in a multimode fiber (1 km) under different parameters (γ = 1, 4, 16, 25, 36). As shown in Fig. R3, the retrieval fidelity using the DENN is quite competitive compared with the one using SSM, further validating the superiority of DENN in realizing optical phase retrieval over a scattering medium.

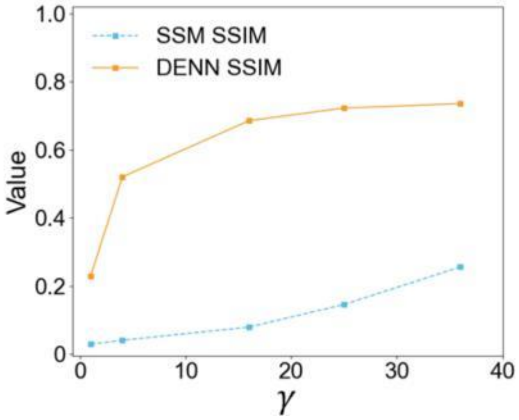

Fig. R3 The experimental results for the dependences of ratio (γ) between the resolutions of the speckle pattern and the phase-encoded information on the performances of the DENN and speckle-correlation scattering matrix methods. The scattering medium is a multimode fiber (diameter is 200 μm, length is 1 km, NA = 0.22, YOFC). The corresponding SSIMs are presented.

The state-of-the-art speckle-correlation scattering matrix method relies on the approximation that the sole eigenvector of the scattering matrix is the retrieval incident field under a sufficiently large γ. While the DENN does not rely on approximation and can achieve better fidelity of optical phase retrieval, as can be seen from Fig. R3. At the same time, general natural scene images that is too diverse to be precisely retrieved by the data-driven deep neural network can be explicitly retrieved utilizing the DENN, where the highest retrieved fidelity is more than 48% better than the data-driven case (30000 data pairs). All these results showcase the superiority of the DENN.

Yes, the Referee is right. It is very important to point out the potential limitation of DENN for optical phase retrieval over a scattering medium. We believe that the current inference time of the DENN is still limited. We have tried to further reduce the inference time from 270s to 90s with the same fidelity by using a more lightweight neural network, where it could be further reduced by optimizing the architecture of the neural network. There is another limitation of DENN, where a sufficiently large ratio (γ) between output and input channel numbers is crucial for guaranteeing a high fidelity of optical phase retrieval. Compressed sensing might be used to reduce the size of speckle required to achieve high fidelity of optical phase retrieval.

In order to address this very important comment, the sentence in Supplementary information is changed from "The SSM method is also based on the empirical TM." to "The SSM method is also based on the empirical TM, where a sufficiently large ratio (γ) between resolutions of the speckle pattern and the encoded information is necessary for achieving high fidelity optical phase retrieval. It relies on the approximation that the sole eigenvector of the scattering matrix is the retrieval incident field under a sufficiently large γ."

The following sentences in Supplementary information is also changed from "In contrast, the retrieved PCC/SSIM of natural scene images using the DENN is as high as 0.97/0.76 and the retrieved accuracy of binary information using the DENN is as high as 99.2% under the same experiment conditions, showing good superiority of the DENN for optical phase retrieval over a scattering medium." to "Because the DENN does not rely on approximation, the retrieved PCC/SSIM of natural scene images using the DENN is as high as 0.97/0.76 and the retrieved accuracy of binary information using the DENN is as high as 99.2% under the same experimental conditions, showing good superiority of the DENN for optical phase retrieval over a scattering medium."

We also add the following sentences "There are still limitations of the proposed DENN. The inference time and the size of speckle pattern required can be further reduced.". We also revise the English presentation, as indicated by the red color in the marked PDF.

In summary, we really thank the Referee for the very important and inspired comments in two rounds of review, which substantially help us to improve the quality of our manuscript and show us the direction of future work. In order to address these comments, we perform new experimental results to further strength the proposed concept and revise the presentation of our manuscript to improve the quality of our manuscript. It is anticipated that the revised manuscript can now meet the criteria of Nature Communications and your consideration is sincerely appreciated.

%%%%%%%%%%%%%%%%%%%%%%%%%%%%%%
Response to the Referee 2
%%%%%%%%%%%%%%%%%%%%%%%%%%%%%%
Blue color outlines the original text
Red color indicates the corresponding modifications

GENERAL COMMENT 1:
I thank the authors for carefully addressing my comments by providing clear explanations, revising the manuscript and title, and conducting extra experiments. The quality and clarity of the revised manuscript has been significantly improved, and most of my previous concerns have been sufficiently addressed. Before recommending the manuscript for publication, I have three minor comments to be discussed with the authors.

OUR REPLY:
We really thank the Referee for the very important comments in two rounds of review, which help us to substantially improve the quality of our manuscript. We also would like to thank the Referee for acknowledging "The quality and clarity of the revised manuscript has been significantly improved".

We will carefully address three minor comments raised from the Referee one by one in the following.

TECHNICAL COMMENT 1:
["However, the analytical models can only be applied in the scenario where optical scattering is absent."] To avoid potential misinterpretations, the authors are suggested to revise this sentence because analytic models for strong scattering have been investigated in the context of diffraction tomography, see [1,2].

OUR REPLY:
We would like to thank the Referee very much for pointing out this confused statement. Yes, the Referee is right. These references mentioned by the Referee are very related that we did miss in our previous manuscript. We read these two papers carefully and find that they used beam propagation methods to calculate the estimated diffraction pattern in evaluating the loss function of physical consistency. As mentioned in Ref. [2], "strongly scattering objects typically also exhibit strong back-scattering, which is not mathematically treated in our MSBP model." (Here MSBP is multi-slice beam-propagation), these methods take multiple forward scattering events into account and does not account for backward scattering.

In order to address this very important comment, we change the sentence from "However, the analytical models can only be applied in the scenario where optical scattering is absent[33,34]. There are many optical systems that lack of analytical theory[52], where wave scattering systems are typical examples." to "Although beam propagation method[52] can account for weak scattering scenarios with negligible backward scattering[53,54], the analytical models can only be applied in the scenario where strongly optical scattering is absent[33,34]. Furthermore, there are many optical systems without

analytical theory[55], where wave scattering systems with non-negligible backward scattering are typical examples."

The following references are added:

[52] Roey, J. V., van der Donk, J. & Lagasse, P. E. Beam-propagation method: analysis and assessment. J. Opt. Soc. Am. 71, 803-810 (1981).
[53] Kamilov, U. S. et al. Learning approach to optical tomography. Optica 2, 517-522 (2015).
[54] Chowdhury, S. et al. High-resolution 3D refractive index microscopy of multiple-scattering samples from intensity images. Optica 6, 1211-1219 (2019).

TECHNICAL COMMENT 2:

[Regularization by Denoising] The RED method compared in the experiments is not the exact algorithm proposed in [3]. In fact, the so-called RED method is a combination of RED and DIP. The authors are suggested to rename the method to better reflect this difference.

1. A Learning approach to optical tomography.
2. High-resolution 3D refractive index microscopy of multiple-scattering samples from intensity images.
3. prDeep: Robust Phase Retrieval with a Flexible Deep Network.

REPLY:

We would like to thank the Referee for this very important comment. We are sorry about the confusion we made in previous revision. The RED based method we used in previous revision is the combination of DENN and RED with a pretrained denoiser. We have found that we did not add noise in the speckle when we simulated the effect of applying the methodology proposed in prDeep to improve the performance of the DENN. To provide a quantitative comparison, we perform two new experiments using a 1 km multimode fiber and a ground glass to show the compatible functionality of prDeep to the proposed DENN, where the noise naturally exists in experiments. The new experimental results will be presented in the Reply to Technical Comment 3 in the following.

Follow the suggestion of the Referee, we change the sentences from "We also study the integrating of the empirical model to the self-supervised learning model used in homogeneous media[25] and regulation by denoising[66, 67] (Supplementary Notes 9 and 10), respectively, where the results are not as good as the DENN." to "We also study the integration of the empirical model to the self-supervised learning model used in homogeneous media[25] and prDeep[69] with a pretrained denoiser[70] (Supplementary Notes 9 and 10), respectively."

The sentence "Future efforts that combine the advantages of self-supervised learning model[25], regulation by denoising[69, 70], plug-and-play priors[71] and DENN can be used to further improve the fidelity of optical phase retrieval over scattering media." is changed to "Future efforts that combine the advantages of self-supervised learning model[25], prDeep[69] with a pretrained denoiser[70], plug-and-play priors[71] with the DENN can be used to further improve the fidelity of optical phase retrieval over scattering media."

The sentences in the supplementary material are changed from "We also quantitatively compare performances between the regularization by denoising (RED)[6] and the DENN in simulation. First, we generate the training dataset by adding Gaussian white Noise (std = 30 dB) to 10,000 images from ImageNet3. We get the denoiser by utilizing DnCNN7 to fit the training dataset. The $SSIM_{loss}$ and AdamW are still used as the loss functions and optimizer to minimize the following expressions:

$_{*}(,) = \arg\min_{\tilde{\varphi},m}$ where F^{1}_{m} $\| |\cdot{}^{1}() \cdot |^{2} - \|^{2} + {}^{2}\tilde{}\tilde{(} - {}^{2}{}_{*}\tilde{(})))$ is our neural network mapping the speckle intensity I to the phase $\tilde{}$, X is the RED regularization strength, and F^{2}_{m*} is the pre-trained denoising model. The corresponding comparison results are shown in supplementary Figure 9." to "We also quantitatively study performances of DENN with and without regularization by denoising utilizing a pretrained denoiser[6] in two experiments. First, we generate the training dataset by adding Gaussian white Noise (std = 30 dB) to 10,000 images from ImageNet[3]. We get the denoiser by utilizing DnCNN[7] to fit the training dataset. The $SSIM_{loss}$ and AdamW are still used as the loss functions and optimizer to minimize the following expressions:

$$_{*}(,) = \arg\min_{\tilde{\varphi},m} \| |\cdot() \cdot |_{2} - \|^{2} + {}^{2}\tilde{}\tilde{(} - R_{*}\tilde{(}))$$

where F_{m} is our neural network mapping the speckle intensity I to the phase $\tilde{}$, X is the RED regularization strength, and R_{m*} is the pre-trained denoising model. The corresponding results are shown in supplementary Figure 9."

TECHNICAL COMMENT 3:

To further strengthen the result, a comparison between DENN and the exact algorithm in [3] is recommended, because the recovery performance reported in [3] seems not to be consistent with what has been reported in the revised manuscript.

OUR REPLY:

We would like to thank the Referee for this very important comment. The algorithm in [3] indeed is an elegant method that is fully compatible with the proposed DENN. It is a method that can improve the performance of the DENN rather than a competitive one. Without the empirical model, the prDeep cannot realize the optical phase retrieval presented in this manuscript.

As suggested by the Referee, the performance of DENN can be further improved using the prDeep with a pretrained denoiser. To address this comment, we study the performances of DENN with and without regularization by denoising utilizing a pretrained denoiser. As can be seen in Fig. R4, the fidelity of optical phase retrieval over two scattering media can be further improved using suitable regularization strength (X).

In order to address this very important comment, the sentence "With the method proposed in prDeep[69], the fidelity of optical phase retrieval can be further improved using suitable regularization strength (Supplementary Note 10)." is added.

[Image redacted]

Fig. R4 Performances of DENN with and without regularization by denoising utilizing a pretrained denoiser in two experiments: a 1 km MMF (a) and a ground glass (b). The ground truths, the captured speckle patterns of the MMF/ground glass and the retrieved information of DENN with and without regularization by denoising (RED) utilizing a pretrained denoiser are provided, respectively. Here, two cases of RED (= 0.05, 0.1) are provided. These images are adapted from the ImageNet dataset[3], where they are all 256-level gray scale images with a resolution of 64×64. Their corresponding resolutions of the speckle patterns are 384×384. The corresponding PCC/SSIM is presented. Colorbars are also provided.

The title of Supplementary Note 10 is changed from "Comparison of performances between the regularization by denoising methods and the deep empirical neural network" to "Performances of deep empirical neural network with and without regularization by denoising utilizing a pretrained denoiser".

The sentence "The performances of optical phase retrieval (X = 0.05, 0.1, 0.2) are degraded compared with our case (X = 0)." is changed to "The performance of DENN can be improved using suitable regularization strength (X)."

The supplementary Figure 9 is replaced by the Fig. R4.

The caption of Supplementary Fig. 9 is changed from "Comparison of performance between the regularization by denoising and the DENN in simulation. The ground truths, the estimated output speckle patterns of the MMF and the retrieved information of DENN alone as well as the

combination of DENN and RED are provided, respectively. Here, three cases of RED (λ = 0.05, 0.1, 0.2) are provided. These images are adapted from the ImageNet dataset[3], where they are all 256-level gray scale images with a resolution of 64X64. Their corresponding resolutions of the speckle patterns are 384X384. The corresponding PCC/SSIM is presented. Colorbars are also provided." is changed to "Performances of DENN with and without regularization by denoising utilizing a pretrained denoiser in two experiments: a 1 km MMF (a) and a ground glass (b). The ground truths, the captured speckle patterns of the MMF/ground glass and the retrieved information of DENN with and without regularization by denoising utilizing a pretrained denoiser are provided, respectively. Here, two cases (X = 0.05, 0.1) are provided. These images are adapted from the ImageNet dataset[3], where they are all 256-level gray scale images with a resolution of 64X64. Their corresponding resolutions of the speckle patterns are 384X384. The corresponding PCC/SSIM is presented. Colorbars are also provided."

We also revise the English presentation, as indicated by the red color in the marked PDF.

In summary, we would like to thank the Referee again for the very important and inspired comments in two rounds of review. These comments substantially help us to improve the quality of our manuscript. It is anticipated that the revised manuscript can now meet the criteria of Nature Communications and your consideration is sincerely appreciated.

%%%%%%%%%%%%%%%%%%%%%%%%%%%%%%%%

Response to the Referee 3

%%%%%%%%%%%%%%%%%%%%%%%%%%%%%%%%

Blue color outlines the original text

Red color indicates the corresponding modifications

- -

GENERAL COMMENT:

The authors made accurate responses to all my comments and suggestions. By adding new experimental results and descriptions, the rigor and completeness of this manuscript has been remarkably improved. Therefore, I suggest this manuscript to be accepted.

OUR REPLY:

We would like to thank the Referee again for the very important and inspired comments in the first round of review. These comments substantially help us to improve the quality of our manuscript. We also revise the English presentation, as indicated by the red color in the marked PDF. It is anticipated that the revised manuscript can now meet the criteria of Nature Communications and your consideration is sincerely appreciated.

Response letters

%%%%%%%%%%%%%%%%%%%%%%%%%%%%%%

Response to the Referee 1

%%%%%%%%%%%%%%%%%%%%%%%%%%%%%%

Blue color outlines the original text,

Red color indicates the corresponding modifications

GENERAL COMMENT:

I would like to thank the authors for their detailed responses to the previous questions. The explanations and extra experiments about the previous questions have been provided. Theoretical analysis and extensive experiments prove the technical correctness and effectiveness of DENN. However, as the authors acknowledge, the DENN proposed in this paper can be regarded as a physically driven DIP method for optical phase retrieval, which can still align with previous DIP frameworks for optical phase retrieval. Further, compared to analytical model, the authors have illustrated the advantages and physical significance of empirical model.

OUR REPLY:

We would like to thank the Referee for his/her insightful and constructive comments on our manuscript in two rounds of review, which have substantially helped us to improve the quality of our manuscript.

%%%%%%%%%%%%%%%%%%%%%%%%%%%%%%%%

Response to the Referee 2

%%%%%%%%%%%%%%%%%%%%%%%%%%%%%%%%

Blue color outlines the original text

Red color indicates the corresponding modifications

GENERAL COMMENT:

I thank the authors for further addressing my previous concerns. I have no more comments on the manuscript.

OUR REPLY:

We would like to thank the Referee for his/her insightful and constructive comments on our manuscript in two rounds of review, which have substantially helped us to improve the quality of our manuscript.
